# Constant High-Voltage Triboelectric Nanogenerator with Stable AC for Sustainable Energy Harvesting

**DOI:** 10.3390/mi16070801

**Published:** 2025-07-09

**Authors:** Aso Ali Abdalmohammed Shateri, Salar K. Fatah, Fengling Zhuo, Nazifi Sani Shuaibu, Chuanrui Chen, Rui Wan, Xiaozhi Wang

**Affiliations:** 1 College of Information Science and Electronic Engineering, Zhejiang University, Hangzhou 310027, China; asoali202@zju.edu.cn (A.A.A.S.); fenglingzhuo@zju.edu.cn (F.Z.); zuhudu@zju.edu.cn (N.S.S.); 12431025@zju.edu.cn (C.C.); wanrui@zju.edu.cn (R.W.); 2 Department of Physics, College of Education, University of Garmian, Kalar 46021, Iraq; salarkarim@garmian.edu.krd

**Keywords:** triboelectric nanogenerator (TENG), horizontal motion harvesting, energy conversion, constant-voltage output, alternating current (AC)

## Abstract

Triboelectric nanogenerators (TENGs) hold significant potential for decentralized energy harvesting; however, their dependence on rotational mechanical energy often limits their ability to harness ubiquitous horizontal motion in real-world applications. Here, a single horizontal linear-to-rotational triboelectric nanogenerator (SHLR-TENG) is presented, designed to efficiently convert linear motion into rotational energy using a robust gear system, enabling a high voltage and reliable full cycle of alternating current (AC). The device features a radially patterned disk with triboelectric layers composed of polyimide. The SHLR-TENG achieves a peak-to-peak voltage of 1420 V, a short-circuit current of 117 µA, and an average power output of 41.5 mW, with a surface charge density of 110 µC/m^2^. Moreover, it demonstrates a power density per unit volume of 371.2 W·m^−3^·Hz^−1^. The device retains 80% efficiency after 1.5 million cycles, demonstrating substantial durability under mechanical stress. These properties enable the SHLR-TENG to directly power commercial LEDs and low-power circuits without the need for energy storage. This study presents an innovative approach to sustainable energy generation by integrating horizontal motion harvesting with rotational energy conversion. The compact and scalable design of the SHLR-TENG, coupled with its resilience to humidity (20–90% RH) and temperature fluctuations (10–70 °C), positions it as a promising next-generation energy source for Internet of Things (IoT) devices and autonomous systems.

## 1. Introduction

The rapid advancement of implanted medical devices, artificial intelligence (AI), and the Internet of Things (IoT) has created a significant need for ubiquitous, portable, and decentralized electrical energy. In recent years, the triboelectric nanogenerator (TENG) has gained considerable attention as a viable alternative to conventional energy-harvesting methods. By utilizing the mechanisms of contact electrification and electrostatic induction, the TENG effectively converts mechanical energy from the surrounding environment into electrical energy. TENGs have emerged as a significant alternative for enhancing diverse domains, including energy science, AI, IoT [1,2], environmental preservation, self-sustaining sensors [3,4], wearable technology [5,6,7,8], robotics, and medical science. As vital elements in self-powered systems, TENGs must exhibit exceptional output performance to satisfy the growing requirements of these technologies [9,10,11,12,13]. A particularly innovative application of TENGs lies in their ability to harness energy from friction, derived from the Greek word tribos, meaning friction. While the triboelectric phenomenon has been known for centuries, practical applications were previously limited by poor energy-conversion efficiency [10,14]. Since their introduction in 2012, TENGs have undergone significant development, enabling the efficient capture of various mechanical energies that are abundant in the environment but often go unused [15,16,17,18]. Notwithstanding being known for thousands of years, the low energy-conversion efficiency of the triboelectric phenomenon previously hindered its practical use [19,20,21]. However, since their introduction in 2012, TENGs have undergone significant and accelerated development, paving the way for efficiently capturing various mechanical energies, which are abundant yet often go unused in our environment [22,23,24,25]. TENGs function by the complementary effects of triboelectrification and electrostatic induction. This entails the transfer of charge between two materials exhibiting opposing tribopolarity, emphasizing the need to choose appropriate materials to augment the triboelectric effect and optimize output. Over time, TENGs have developed into four different operating modes, all of which depend on contact electrification, yielding surfaces that are oppositely charged. Subsequently, during relative motion, electrostatic induction drives the conversion of mechanical energy into electrical energy [26,27,28,29]. Fundamentally, TENGs function as variable capacitive electric-field sources, with their output power being directly proportional to the square of the triboelectric charge density. Consequently, enhancing TENG performance primarily revolves around increasing the generated triboelectric charge. The greater the electron affinity difference between two materials, the more significant the triboelectric charge generated [28,29,30,31,32,33]. Among the various modes of TENG operation, the sliding mode has garnered significant attention due to its ability to achieve enhanced electrical performance. Unlike simple contact, the sliding motion between two objects results in a larger magnitude of electric charge transfer across their surfaces [22,32,34]. Additionally, the development of disk-based TENGs demonstrates the effective use of spinning mechanical energy, with these systems powered by the rotating motion of two coaxial disks. Contact electrification occurs when both disks come into contact, and the rotary-sliding motion facilitates electron transfer between electrodes with complementary shapes [15,22,30,35,36].

Disk-based TENGs operating in sliding mode and driven by rotational forces present distinct advantages in terms of electrical performance, whereas contact-separation TENGs are primarily dependent on linear forces [21,37]. A key advantage of TENGs in this configuration is their ability to generate consistent and uninterrupted output power. In contrast, contact-separation TENGs generate a brief but powerful power spike, which may complicate power-management circuit design. However, disk-based TENGs are primarily limited to generating rotating force, which is less abundant than linear mechanical energy. A limited body of research has investigated the operational characteristics of disk-based TENGs functioning in a sliding mode, which harness linear mechanical forces from sources such as wind and water [10,14,19,20,21]. Empirical evidence suggests that fans can play a critical role in transforming linear forces into rotational forces [3,4,5,6]. This study leverages the force-conversion mechanism to enhance the application potential of TENG [14,19,21].

Recent studies have explored the dynamics of TENG operation more deeply, investigating the mechanical–electrical coupling and vibration characteristics that influence charge-transfer efficiency and stability. For instance, research highlights the significance of vibration modes and system dynamics in optimizing TENG performance under varying mechanical inputs [38,39]. Meanwhile, the literature presents modeling approaches that analyze the interplay between mechanical deformation and charge generation, providing insight into designing more efficient TENG structures [40]. These advances have led to refined device architectures and enhanced power output through tailored surface microstructures and optimized motion trajectories.

Moreover, innovative strategies such as hybridization with electromagnetic generators, interface engineering, and machine-learning-assisted material design have further improved the energy-conversion efficiency and long-term operational stability of TENGs [7,25,41,42,43,44,45]. Among the operating modes, the sliding mode is particularly effective due to extended contact time and increased contact area, which facilitates greater charge transfer [25,46,47,48]. Overall, the integration of advanced mechanical analysis and materials engineering underscores the ongoing evolution of TENG technology toward practical, high-performance energy-harvesting devices.

This research introduces a novel disk-shaped triboelectric nanogenerator (TENG) designed to effectively harvest linear mechanical energy from both human and machine motions. The system comprises two identical disks that provide constant voltage and an alternating current (AC). A single horizontal-to-rotational triboelectric nanogenerator (SHLR-TENG) was experimentally developed to convert horizontal forces into rotational motion through a precisely engineered gear system, where copper (Cu) slides against various polymer materials (PMs). The study investigates the performance differences among these materials, revealing that the choice of PMs significantly influences the output open-circuit voltage (Voc) and short-circuit current (Isc). Comparative analysis demonstrates that certain PMs produce higher Voc and Isc values, resulting in enhanced power output. Additionally, the ability to maintain constant voltage and current is another novel aspect of this research. Additionally, the system demonstrates exceptional endurance, operating reliably over 1.5 million cycles. This highlights substantial progress in performance optimization and energy conversion, ensuring long-term reliability and stability. The completion of a complete AC cycle optimizes energy-harvesting efficiency and stabilizes the rectified direct current (DC) output. This reduces fluctuations while connecting LEDs to a value that may be deemed inconsequential. This enhances the system’s versatility across many applications. The TENG system’s exceptional durability renders it advantageous for practical applications, providing effective energy conversion and extended operation, thus presenting a feasible solution for various energy-harvesting requirements. Consequently, the SHLR-TENG is capable of powering tiny electronic devices, such as light-emitting diodes (LEDs) and capacitors.

## 2. Materials and Methods

### 2.1. Design and Fabrication of the Triboelectric Nanogenerator (Rotator and Stator)

The triboelectric nanogenerator (TENG) components, including the rotator and stator, were designed using SolidWorks (Version 34.1) and subsequently fabricated by a manufacturer (Shenzhen, China). The substrate was constructed from light-green glass epoxy, with a thickness of 1.5 mm. The rotator and stator had a radius of 7.5 cm and 8 cm, respectively. A central aperture of 0.25 cm radius was incorporated into the rotator to enable smooth rotation along a fixed axis, facilitating energy transmission. Similarly, the stator featured a central aperture with a radius of 0.75 cm.

The substrate’s surface was patterned with copper (Cu), forming the essential conductive elements. The rotator surface was designed with 19 copper sectors, while the stator incorporated 38 copper sectors, as depicted in the enlarged images in Supplementary Figure S1. The copper patterns on the stator formed two electrodes (Electrode 1 and Electrode 2), whereas the copper patterns on the rotator functioned solely as a friction layer to enable contact-electrification with the polymer material (PM) films on the stator. Both the stator and rotator were precisely fabricated using a laser-cutting machine to ensure accuracy and consistency in the TENG disk design.

### 2.2. Materials Selection

The triboelectric nanogenerator (TENG) employed a combination of polymer materials and copper (Cu) to exploit their respective triboelectric properties. Copper, known for its high electron-donating capability, was used as the positive triboelectric material, while several negatively charged polymer materials were selected based on their high electron affinity. These polymers included polycarbonate (PC), polyvinyl chloride (PVC), polyimide (PI), polytetrafluoroethylene (PTFE), fluorinated ethylene propylene (FEP), polyetherimide (PEI), and polyethylene terephthalate (PET).

The effective triboelectric charge transfer between copper and these polymer materials enabled high-output voltage generation, a crucial factor for TENG performance. Copper was particularly advantageous due to its excellent electrical conductivity, mechanical strength, and durability. The selected polymer materials provided low friction, strong electron affinity, and chemical stability, making them ideal candidates for TENG applications. Additionally, their cost-effectiveness and ease of fabrication facilitated large-scale production. This material combination proved to be highly suitable for energy-harvesting applications and wearable electronics due to its reliability in generating a stable energy output.

### 2.3. Gear System and Power Transmission

The TENG system utilized a gear-based power transmission mechanism, incorporating four distinct types of gears (G1, G2, G3, and G4) along with a rack gear. All gears were fabricated from blue nylon to ensure lightweight construction and efficient operation. Each gear featured a uniform module, a tooth thickness of 1 cm, and a step height of 0.8 cm. The detailed specifications of the gears were as follows:

G1: 30 teeth, outer diameter of 3.2 cm, inner bore of 1.4 cm.

G2: 80 teeth, outer diameter of 8.2 cm, inner bore of 1.0 cm.

G3: 30 teeth, outer diameter of 3.2 cm, inner bore of 1.0 cm.

G4: 15 teeth, outer diameter of 1.7 cm, inner bore of 0.5 cm.

Rack Gear: Dimensions of 20 × 20 × 200 mm^3^.

The arrangement and design of the gear system are illustrated in Supplementary Figures S2 and S3, providing both two-dimensional and three-dimensional views. The rack gear played a crucial role in power transmission by converting linear motion into rotational motion. The gear G1 was specifically designed to function as a clutch mechanism by integrating a one-way bearing (10 × 12 × 14 mm^3^). This unidirectional bearing allowed G1 to selectively receive power transmitted by the rack gear, ensuring controlled movement. A shaft and lock nut were incorporated to facilitate secure rotation and maintain system stability.

## 3. Result and Discussion

### 3.1. Device and Structural Design

The schematic representation of the SHLR-TENG, activated by linear mechanical force, is presented in Figure 2a. The SHLR-TENG is made up of two main components, an energy-generation unit and a force-conversion unit, both designed in a cubic configuration. Supplementary Figure S4 presents visual representations of the device. In the force-conversion process, a cylindrical button undergoes linear mechanical force, which is applied by a stepper motor and then converted into rotational mechanical force through the gear system. The stepper motor operates by alternating between forward and backward movements to facilitate this transformation. The resulting rotational mechanical energy is transferred directly to the energy-generation unit, ensuring effective energy conversion during the alternating operational phases of the SHLR-TENG. Once the linear force is released from the rack gear, the system returns to its initial position with the help of the stepper motor.

**Figure 1:**
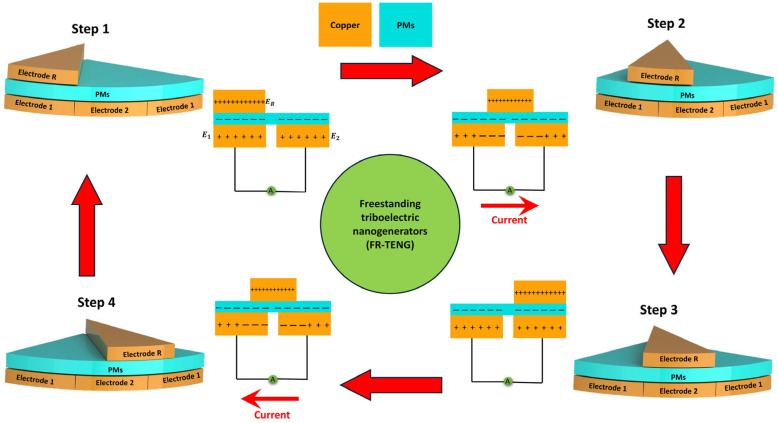
Operating principle of the FR-TENG.

**Figure 2:**
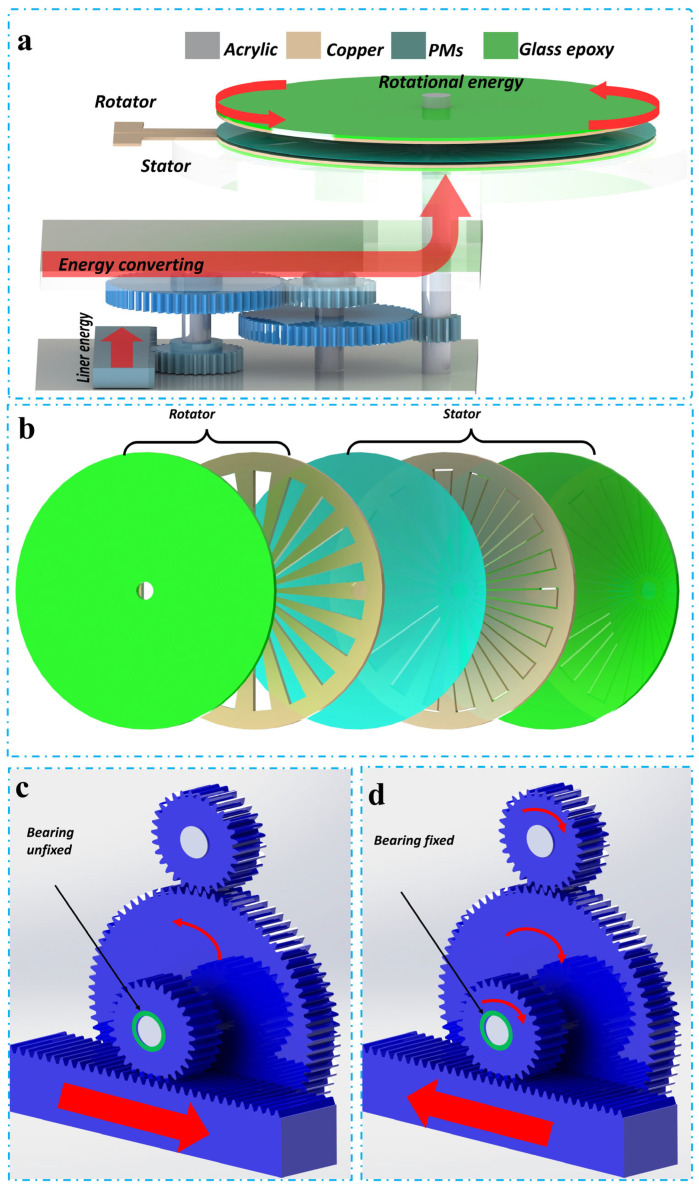
(**a**) A graphical representation of the SHLR-TENG, that includes both a force-conversion section as well as an energy-generation section. (**b**) The 3D layout of the FR-TENG includes a rotator and stator designed from negative triboelectric materials (PMs). Diagrams (**c**,**d**) demonstrate the one-way bearing mechanism, allowing rotation solely in one direction.

Figure 1 shows the operational principle and structural design of the freestanding TENG (FR-TENG). This two-dimensional schematic highlights the stator and rotor components, emphasizing their interaction in the energy-conversion process. Figure 2c,d also depict the one-way bearing mechanism, which permits rotation in only one direction. This mechanism ensures that the generated mechanical energy is efficiently converted into electrical energy while preventing backward motion. Figure 2b presents a three-dimensional exploded view of the FR-TENG, displaying the arrangement of the rotator and stator components constructed from negatively charged triboelectric materials (PMs). The structural design of the FR-TENG is essential for enhancing energy transfer and improving overall system efficiency. The optimized arrangement of the rotor and stator, made from triboelectric materials, maximizes the interaction between the components, increasing charge generation. Strategic electrode placement ensures efficient capture and conversion of mechanical energy into electrical energy. This well-designed structure minimizes energy losses and maximizes the mechanical-to-electrical energy conversion, significantly boosting the system’s efficiency for energy-harvesting applications [49,50,51,52].

**Figure 3:**
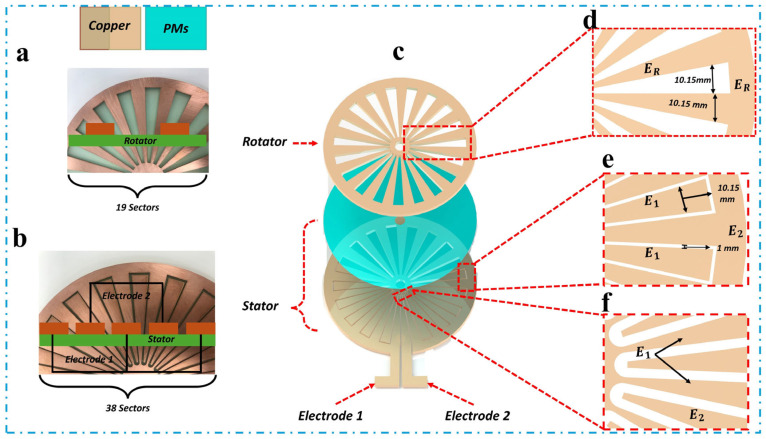
Structural configuration of the energy-generating component. (**a**) The rotator’s surface pictures show 19 Cu sectors. (**b**) Surface pictures of the stator, with 38 patterned Cu sectors distributed over its surface. (**c**) A three-dimensional diagram of the system, depicting the alignment of the rotator (upper layer) and stator (lower layer), while emphasizing the electrical terminals (Electrode 1 and Electrode 2). (**d**,**e**) Detailed close-ups of the external extremities of the rotator and stator, whereas (**f**) illustrates the internal extremity of the stator, demonstrating that the two electrodes possess complementary configurations, divided by narrow trenches.

As depicted in Figure 3, the SHLR-TENG employs a multilayered structure consisting of both a rotator and a stator. The substrate for each disk is made from a light-green glass epoxy material, with copper patterns on its surface. The rotator is composed of 19 copper sectors, while the stator consists of 38 copper sectors arranged radially to ensure optimal interaction during operation (Figure 3a,b). Each sector in the design has an outer length of 5.5 cm and a central angle of 9.474°. The external spacing between the rotator sectors is 1.115 cm, and the electrode spacing on the stator is 1 mm, as outlined in Figure 3d,e. The stator incorporates two electrode sets (Electrode 1 and Electrode 2), with each set comprising 19 sectors separated by narrow trenches. Figure 3f demonstrates the integrated electrodes in the stator, which feature a fixed digitiform configuration with a curved geometry. Additionally, the device incorporates polymer materials (PMs) between the layers, as shown in Figure 3c, ensuring structural integrity and long-term stability. This design enhances assembly efficiency and operational longevity, making the TENG suitable for sustained energy-harvesting applications.

### 3.2. The Development and Evaluation of SHLR-TENG: Mechanisms for Output Optimization and Energy Conversion

**Figure 4:**
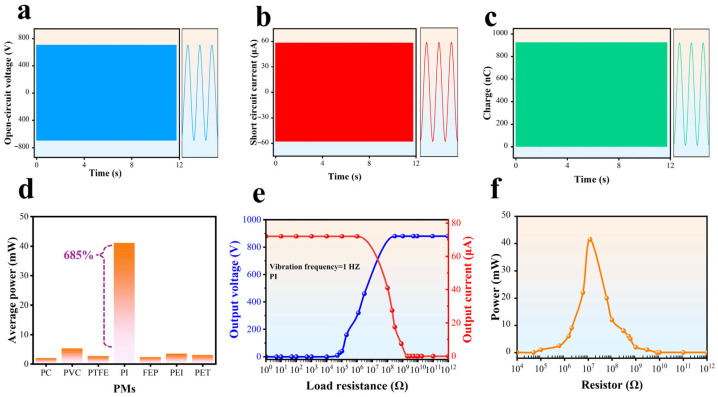
Various electrical characterizations of the SHLR-TENG. (**a**) Waveform of the open-circuit voltage (Voc). (**b**) Waveform of the short-circuit current (Isc). (**c**) Waveform of the short-circuit charge (Qsc). (**d**) Diagram showing the total power output of the TENG across various PMs. (**e**) The relationship between the output voltage, output current, and resistance for the SHLR-TENG. (**f**) The effect of resistance on the output power of SHLR-TENG.

The energy efficiency of the SHLR-TENG is assessed by measuring voltage and current values while monitoring the motion of the rack gear in 6.5 cm increments under both open-circuit voltage (VOC) and short-circuit current (ISC) conditions. Figure 4a,b illustrate the significant voltage and current outputs, which are sufficient to power red LEDs. This highlights the ability of SHLR-TENG to convert linear mechanical energy into electrical energy, making it suitable for powering small electronic devices like capacitors, LEDs, and calculators. However, a critical challenge in the deployment of TENG devices is the impedance mismatch between the power control unit and TENG, leading to reduced power output. Addressing this issue requires an effective optimization strategy to enhance energy-conversion efficiency.

The design of the SHLR-TENG structure is crucial to its overall performance. The device comprises a stator and a rotor, with the stator surface arranged into distinct sectors. Figure 1 provides a three-dimensional view of the FR-TENG configuration, which consists of a rotor and a stator fabricated using copper (Cu) and negatively charged triboelectric polymer materials (PMs). The rotor contains a metal electrode (ER), while the stator is equipped with an insulating sheet, both intended to interact with the electrical terminals on the lateral side of the stator, which is meticulously crafted using laser-cutting technology to enhance the electrical output of the TENG. The ER electrode in the rotor demonstrates electrical buoyancy, whereas the energy-generating component cannot fully capitalize on the retroactive linear force induced by the stepper motor’s restorative force, as shown in Figure 2c,d. This limitation arises due to the unidirectional bearing in the gear system, preventing the effective conversion of force. The stepper motor operates bidirectionally in conjunction with the rack gear, converting linear motion into rotational force. During forward movement, the linear force exerted on the one-way bearing transforms into rotational force. However, the gear system’s one-way bearing fails to engage during forward movement, leading to incomplete force conversion. The absence of a one-way bearing impairs the generation of stable electrical output and restricts the rack gear’s ability to reset to its initial position, thereby limiting its capacity to receive subsequent linear forces in forward motion. The gear system’s complete specifications are detailed in Supplementary Figure S4. The rotational motion of the disk is calculated by multiplying the rotations of gear 1 by the system’s rotation ratio. Gear 1, with a diameter of 3.2 cm, completes approximately 0.70 rotations when the stepper motor moves linearly by 6.5 cm. Given the system’s rotational ratio of approximately 10 rotations, each full revolution of gear 1 results in roughly 10 rotations of the SHLR-TENG rotator. For example, applying a 1 cm linear force results in approximately 1.538 rotations of the rotator disk. In this study, a linear force of 6.5 cm was applied per measurement, leading to nearly 10 revolutions of the rotator within the gear system. Supplementary Note S1 and Supplementary Table S1 show further details on velocity calculations and energy-transfer optimizations.

The SHLR-TENG’s energy-generating mechanism consists of a single rotating disk, known as the rotator, which transfers mechanical rotational force from the energy-conversion component. Simultaneously, the stator remains fixed, secured within the cubic shell. A real-time demonstration of SHLR-TENG’s operation is provided in Supplementary Video S1. Figure 3a,b depict the surfaces of both the rotor and stator, with the substrate made from light-green glass epoxy material, featuring a radius of 7.5 cm and featuring projected copper (Cu) patterns. The enlarged images in Figure 3a,b show that the rotator contains 19 copper sectors, whereas the stator holds 38 radially arranged copper sectors. Each sector has an outermost length of 5.5 cm and a central angle of 9.474°. The spacing distances between the rotator and stator were measured at 1.115 cm and 0.1 cm, respectively. The Cu pattern on the stator is segmented into two electrodes, Electrode 1 and Electrode 2, each featuring 19 sectors arranged in a finger-like pattern. The rotor and stator components were both manufactured with laser-cutting technology, which ensures precise fabrication for efficient energy generation.

The triboelectric layer on the stator consists of a polymer material (PMs) sheet, which interacts with the Cu projections on the rotator through sliding and rotation to enhance electrical output. According to the triboelectric series, after pre-charging, the rotor develops a positive charge, while the PMs layer on the stator gains a negative charge. Electrodes 1 and 2 are connected to a constant-voltage alternating current (AC) source generated by the continuous rotation of the rotator. Figure 4a,b demonstrate the application of Ohm’s Law (V = IR), emphasizing the relationship between voltage (V), current (I), and resistance (R) in the Cu-PMs system. This relationship is key to understanding the time-dependent electrical behavior. When resistance is constant, the voltage across the interface stays proportional to the current in the circuit. However, during sliding motion, both current and voltage exhibit only minimal fluctuations, which are considered negligible. Additionally, Figure 4c demonstrates the QSC of SHLR-TENG during each vibration cycle, with a measured value of 936 nC. This indicates strong charge generation and transfer efficiency, highlighting its potential for energy harvesting from mechanical vibrations. Load resistance significantly impacts output current and voltage, assuming constant mechanical energy input. As shown in Figure 4d,e, the peak current output was measured at 58.5 μA with a load resistance of 500 kΩ, while the voltage remained relatively low because of the device’s high internal impedance. According to Ohm’s Law, as resistance increased beyond approximately 12 MΩ, the current gradually decreased while the voltage increased. The inverse relationship between current and voltage underscores the importance of optimizing load resistance to improve device performance and energy-harvesting efficiency. Selecting an appropriate dielectric layer is crucial in TENG applications. Several dielectric materials, including PET, PTFE, PVC, PC, PI, PEI, and FEP, were extensively evaluated in this study for their potential to optimize power output. Performance comparisons were based on triboelectric properties and energy-conversion efficiency. Following rigorous testing, polyimide (PI) exhibited the highest power output and superior stability, as demonstrated in Figure 4f. Consequently, PI was identified as the optimal dielectric layer for this study, significantly enhancing the overall efficiency of SHLR-TENG.

### 3.3. Electricity-Generating Process

#### 3.3.1. SHLR-TENG’s Output Performance Under a Resistance Load

As depicted in Figure 2b, this device leverages triboelectric materials. It consists of a metal electrode (ER) in the rotor, with an insulating layer on the stator’s surface, along with two separate electrodes (E1 and E2). To guarantee correct alignment and fit, laser-cutting technology was used to produce the rotator and stator with extreme accuracy. The TENG output is made possible by the electrodes’ connection to the electrical terminals. Under VOC, electrons cannot move between the electrodes. The potential difference between the two electrodes is referred to as the (Voc) = VE1 − VE2.

The peak V_OC_ occurs in the first stage when E_1_ obtains its highest potential and E_2_ reaches its lowest potential. The voltage decreases gradually when the rotator starts to rotate. The V_OC_ changes phase once the rotator crosses halfway and continues rising until Step III is achieved. Due to the structure’s periodic nature, the V_OC_ then changes direction. An analytical model can be developed if the thickness of the dielectric layer is much smaller than the width shown in Figure 2b. This model neglects edge effects, assuming that any overlap between the rotor and electrodes behaves like a parallel-plate capacitor. As illustrated in Supplementary Figure S5 and Supplementary Note S2, the open circuit voltage (V_OC_) can be derived analytically through Gauss’s Theorem.

The average power across a resistive load ($P_{R}^{AP}$) could be determined using the subsequent equation:

(1) $$P_{R}^{AP}=\frac{\int_{t}^{t+∆T}I^{2}Rdt}{∆T}$$

where Δ*T* is the measured time, *I* is the experimental current, and *R* is the load resistance. It was observed that with a load resistance of 12 MΩ, PI achieves a highest power of 41.535 mW, as shown in Figure 4d. The maximum average power values for various PMs are also depicted in Figure 4d. At frequencies of 0.5, 1, 2, and 4 Hz, the PMs achieved the highest average power values of 3.30, 6.95, 13.845, and 41.535 mW, respectively. At 4 Hz, the materials PC, PVC, PTFE, FEP, PEI, and PET achieve maximum average power values of 2.03, 5.29, 2.70, 2.35, 3.47, and 3.07 mW, respectively.

In conclusion, under a resistance load, PI consistently outperforms other materials, delivering a significant improvement (7.85-times higher than the maximum value of 5.29 mW seen in other materials). This indicates that using PI as the dielectric layer is a highly effective method for substantially enhancing energy generation in TENG applications.

#### 3.3.2. Output Performance of SHLR-TENG as a Function of Gap Distance Between Stator and Rotor

**Figure 5:**
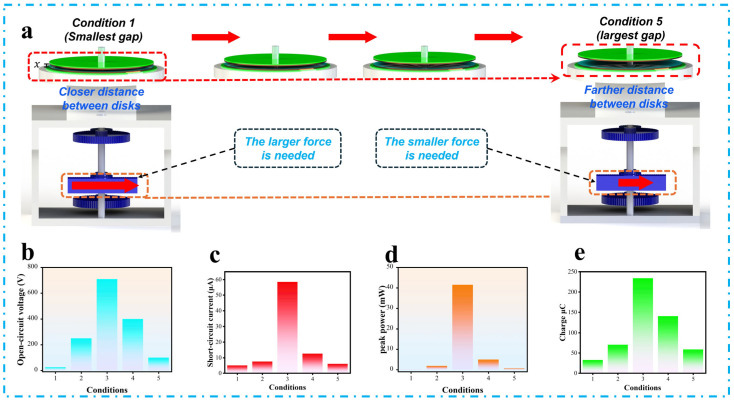
Electrical characterization of the SHLR-TENG for five different conditions of (x): (**a**) Schematic representation of five states of the disk-to-disk distance, (**b**) V_OC_, (**c**) I_SC_, (**d**) average power P_AV_, (**e**) short-circuits charge (Q_SC_).

As illustrated in Figure 5a, the gap distance (x) is adjustable to regulate the downward force needed for sliding. A small x creates high pressure, enabling strong sliding with a larger frictional force. To measure the electrical output of the SHLR-TENG, five different values of x were tested, as a larger x leads to lower pressure, which causes weaker sliding and a smaller frictional force.

By adjusting the separation distance (x) between the rotator and stator, ranging from 0 mm in Condition 1 (smallest gap) to 0.4 mm in Condition 5 (largest gap), the performance of the SHLR-TENG was systematically evaluated across five distinct conditions. As the separation distance increased linearly across all scenarios, smaller distances resulted in stronger sliding due to higher frictional forces, though they required more mechanical effort to operate the device efficiently.

In contrast, larger x values reduced friction, leading to weaker sliding, which made pressing the button easier but negatively impacted energy-production efficiency. As depicted in Figure 5b–d, Condition 3 yielded the highest electrical outputs for the SHLR-TENG with VOC of 710 V, ISC of 58.5 μA, and an average power (PAV) of 41.535 mW, respectively. Furthermore, Figure 5e illustrates the QSC of the SHLR-TENG resulting from each cycle of vibration. To directly compare an ISC value with its equivalent QSC, the QSC value was computed using the integral of ISC over the time interval. The QSC trend under each scenario mirrors that of VOC and ISC. In Condition 3, the peak QSC value was approximately 936 nC, which corresponds to the QSC transmitted by the SHLR-TENG during one vibration cycle.

This condition was identified as having the optimal x value. Beyond Condition 3, lower x values caused excessive friction, increasing mechanical effort and reducing electrical output, while larger x values resulted in weaker sliding, decreasing layer contact and lowering performance. This behavior highlights the importance of finding a balance between mechanical force and energy-generation efficiency, with a clear inverse relationship between sliding efficiency and separation distance (x). The findings underscore the critical role of fine-tuning the separation distance (x) to optimize the SHLR-TENG’s energy-harvesting efficiency, making it a viable energy-generation solution.

#### 3.3.3. SHLR-TENG’s Output Performance Under a Capacitance Load

**Figure 6:**
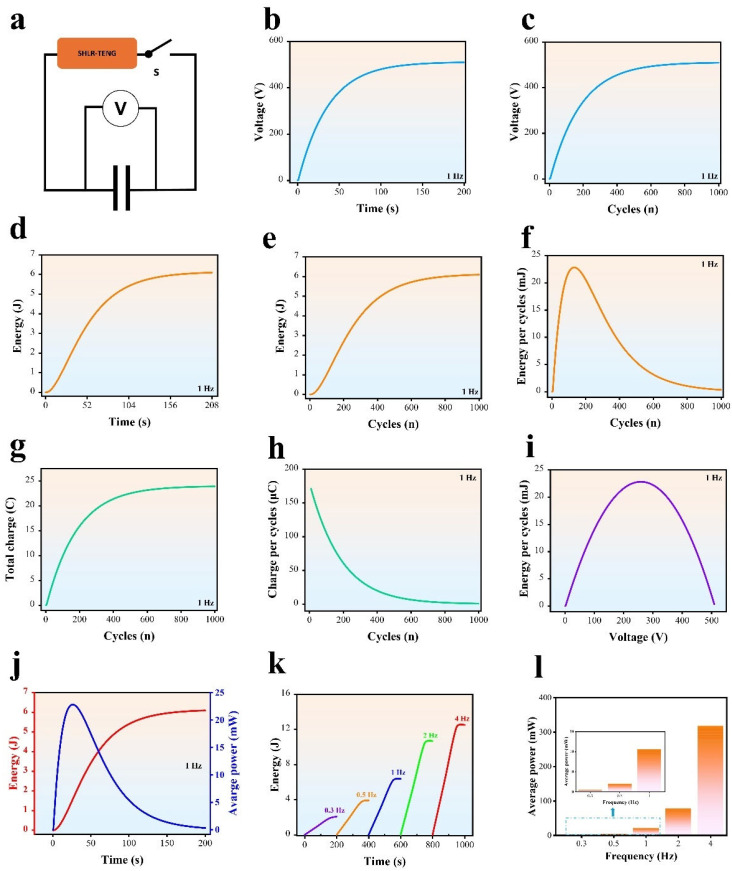
The performance of the SHLR-TENG when connected to a capacitive load. (**a**) The circuit configuration linking the TENG and the capacitor. (**b**) The voltage across the capacitor during charging. (**c**) The variation in capacitors charging voltage per cycle. (**d**) The capacitor’s stored energy over time. (**e**) The total energy transferred per cycle. (**f**) The energy transferred to the capacitor per cycle as a function of the number of cycles when charged by the SHLR-TENG. (**g**) The total amount of charge transferred to the capacitor per cycle. (**h**) The charge transferred to the capacitor per cycle plotted against the number of cycles. (**i**) The energy transferred to the capacitor per cycle compared with the voltage across the capacitor when charged by the SHLR-TENG. (**j**) The stored energy and the average power variation over time during charging by the SHLR-TENG. (**k**,**l**) The variation in the stored energy of the capacitor over time when charged by the SHLR-TENG at different charging conditions.

The exceptional output capability of TENGs is essential for their effective operation as a widespread power source or key component in self-sustaining technologies. Since all current power-management methods rely on capacitors to initially harvest electrical energy from the TENG, it is crucial to thoroughly assess the performance of SHLR-TENGs under capacitive loads in order to maximize the average power output. In this study, the performance of SHLR-TENGs under capacitive load is evaluated using a single 47 μF capacitor. The SHLR-TENG, which is controlled by switches (S), is connected to the capacitor as shown in Figure 6a. Figure 6b,c present the relationship between the capacitor’s voltage and time, as well as the number of cycles, respectively. Figure 6d,e show how the energy stored in the capacitor evolves over time and cycles, respectively. Figure 6g depicts the total charge accumulated in the capacitor as a function of cycles. Additionally, Figure 6f,h illustrate the charge transferred to the capacitor per cycle in relation to the cycle number, as well as the energy supplied to the capacitor per cycle at a constant mechanical frequency of 1 Hz, respectively. For detailed information, see Supplementary Note S4. The voltage over the capacitor charged by the SHLR-TENG consistently rises to 510 V, signaling that the TENG reaches its maximum charging rate after several cycles, as seen in Figure 6c. The energy production from SHLR-TENG consistently grows and reaches its highest level with the increase of cycles. After just 1000 cycles, the SHLR-TENG stores 6.13 J, as seen in Figure 6e.

Furthermore, the charge that flows to the capacitor during each cycle is an essential factor for assessing the electric-quantity output capabilities of a TENG when subjected to a capacitance load, much like the way average current serves to standardize the electric-quantity output of a TENG under a resistance load. Figure 6h illustrates that the SHLR-TENG demonstrates remarkable efficiency in transferring charge to the capacitor, consistently achieving a maximum charge of 172.35 µC per cycle after only 1000 cycles. Additionally, Figure 6g presents the total charge accumulated in the capacitor over the cycles. The energy output of the SHLR-TENG under a capacitance load shows a significant increase, equivalent to the rise in electrical quantity. The energy delivered to the capacitor per cycle for the SHLR-TENG reaches a peak of 23.9 mJ, showcasing the distinctive advantages of the SHLR-TENG regarding energy output, as illustrated in Figure 6f.

Additionally, a strong correlation exists between the energy delivered to the capacitor per cycle and its voltage. The energy output per cycle escalates with rising voltage up to the point where it reaches the maximum average power, thereafter declining, as seen in Figure 6i. Hence, it is important to determine the optimal resistance for a TENG under a resistive load to maximize average power output, and to identify the corresponding capacitor voltage for a TENG under a capacitive load for optimal performance. The instantaneous power ($P_{ins}^{C}),$ instantaneous energy ($E_{ins}^{C}$), average power ($P_{AP}^{C}$), energy flowing to the capacitor per cycle $E_{n}^{C}$, and charge flowing to the capacitor per cycle $Q_{n}^{C}\;$ of a capacitor can be represented using the following equations:

(2) $$P_{ins}^{C}=U_{C}i={CU}_{C}\frac{{dU}_{C}}{dt}$$

(3) $$E_{ins}^{C}=\int_{0}^{T}P_{ins}^{C}dt=U_{C}i=\int_{0}^{T}{CU}_{C}\frac{{dU}_{C}}{dt}\;dt=\frac{{CU}_{C}^{2}}{2}$$

(4) $$P_{AP}^{C}=\frac{E_{ins}^{C}}{T}\frac{{CU}_{C}^{2}}{2T}$$

(5) $$Q^{C}=CU_{C}$$

(6) $$E_{n}^{C}=\frac{1}{2}\;CU_{C}\;{\left(n\right)}^{2}-\frac{1}{2}CU_{C}\;{\left(n-1\right)}^{2}\;$$

(7) $$Q_{n}^{C}=CU_{C}\;(n)-CU_{C}\;\left(n-1\right)$$

In this context, *C* represents the capacitance of the capacitor, *Q^C^* signifies the total charge stored in the capacitor, *U_C_* indicates the voltage across the capacitor, n represents the cycle number, and *T* refers to the charging time. Furthermore, the connection between the average power over time and energy stored is illustrated in Figure 6j. The SHLR-TENG showcases impressive energy-output performance, even at the minimal frequency of 1 Hz. Conversely, Figure 6k demonstrates the total energy-storage amounts under a capacitance load at various frequencies. The SHLR-TENG attains impressive energy-storage values of 2.1, 3.67, 6.13, 10.4, and 12.05 J at frequencies of 0.3, 0.5, 1, 2, and 4 Hz, respectively. Likewise, the peak average power values for a capacitance load at these frequencies are represented in Figure 6l. The SHLR-TENG demonstrates noteworthy average power outputs of 0.9, 3.97, 22.8, 77.62, and 318 mW at frequencies of 0.3, 0.5, 1, 2, and 4 Hz, respectively. The resultant performance highlights SHLR-TENG’s capability of generating substantial energy and power, even at low operational frequencies.

The resistance load with energy output allows for the investigation of the highest energy output of a TENG. The energy output with a capacitive load facilitates the identification of an optimal power-management technique to maximize energy output.

**Figure 7:**
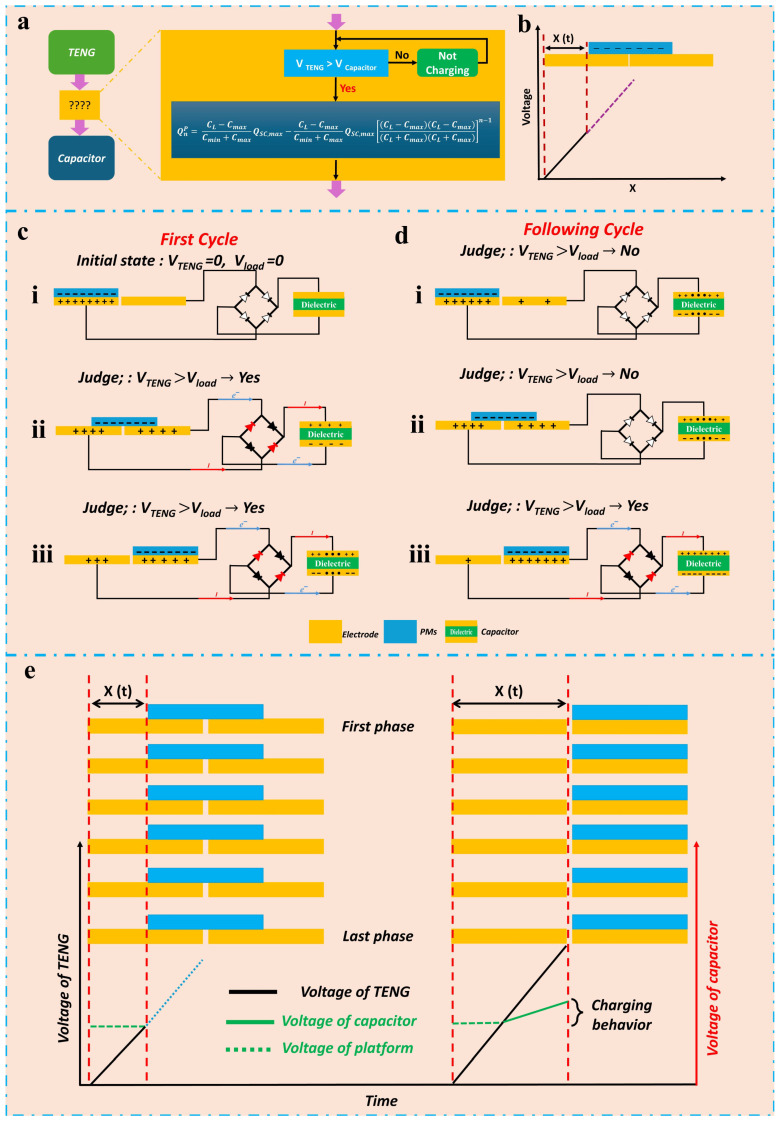
The dynamic charge-transfer process under a capacitive load. (**a**) A diagram depicting the dynamic behavior of a TENG while charging a capacitor. (**b**) The variation in TENG voltage in relation to the motion path during free-standing mode. Logical representations of (**c**) the initial cycle and (**d**) subsequent cycles for a TENG charging a capacitor. (**e**) Schematic illustrations of the charging mechanisms of the SHLR-TENG under a capacitive load.

The analysis of the dynamic charge-transfer process under a capacitive load is presented to highlight the superior output performance and investigate the underlying factors that enable the SHLR-TENG to enhance energy output and electrical characteristics, as explained by the electrical principles of TENGs and shown in Figure 7. Figure 7a illustrates that modeling the dynamic process of a TENG charging a capacitor requires focusing on real-time performance during each cycle, emphasizing the importance of monitoring both the TENG capacitor voltage and the overall voltage during dynamic operation. Charging behavior takes place only when the real-time TENG voltage exceeds that of the capacitor. Typically, under short-circuit conditions or a resistive load, it generates a steady amount of electric charge in each cycle, driven by the coupling effects of contact electrification and electrostatic induction. However, this behavior changes once the TENG starts charging the capacitor, as the charge produced through contact electrification and electrostatic induction can be categorized into two types: transferred charge (TC) and reserved charge (RC). TC is utilized to charge the capacitor, whereas RC is essential for sustaining the voltage between the TENG and the capacitor. The electric quantity ($Q_{n}^{P}$) of TC can be derived as follows [53,54]:

(8) $$Q_{n}^{P}=\frac{C_{L}-C_{max}}{C_{min}+C_{max}}Q_{SC,max}-\frac{C_{L}-C_{max}}{C_{min}+C_{max}}Q_{SC,max}{\left[\frac{\left(C_{L}-C_{max})(C_{L}-C_{max}\right)}{\left(C_{min}+C_{max})(C_{min}+C_{max}\right)}\right]}^{n-1}$$

where *C_L_* is the capacitance of the capacitor, $Q_{n}^{P}$ is the charge on node *P* at the time of the *n*th cycle, *C_max_* and *C_min_* are the maximum and minimum values of the variable capacitance of the TENG, respectively, and *P* is a node on the circuit of the TENG charging the capacitor. Detailed information is provided in Supplementary Figure S7 and Supplementary Note S5. 

As presented in Figure 7d, the ratio of TC diminishes as the capacitor voltage rises, aligning with the experimental data. The aforementioned dynamic method is applicable to all categories of TENGs, as unique category information is not included in this process.

Figure 7b presents a simple model demonstrating the correlation between the motion direction and TENG voltage, using free-standing mode as an instance. Figure 7c,d depicts a model incorporating a TENG that charges a capacitor via a rectifier, showcasing the relationship between the charging behavior and motion direction in each cycle.

As illustrated in state (i) in Figure 7c, during the initial cycle, a quasi-charging state occurs where the voltages of capacitors and TENG are at zero. As the TENG begins to move, a charging process begins, which continues until the TENG’s voltage reaches its peak as shown in (states (ii) and (iii) in Figure 7c). Charging behavior is missing in subsequent cycles in state (i), as illustrated in Figure 7d, since the TENG voltage is less than the capacitor voltage. As the TENG begins to move, it is essential to determine if the voltage of the TENG exceeds that of the capacitor, as illustrated in state (ii) in Figure 7d. If the answer is no, then charging behavior does not occur. Should the answer be affirmative, charging behavior continues until state (iii) in Figure 7d is attained. Meanwhile, the integrated model is constructed to illustrate the progression of the SHLR-TENG from the initial phase to the final phase, as depicted in Figure 7e.

### 3.4. Durability and Reliability of SHLR-TENG Under Environmental Conditions

**Figure 8:**
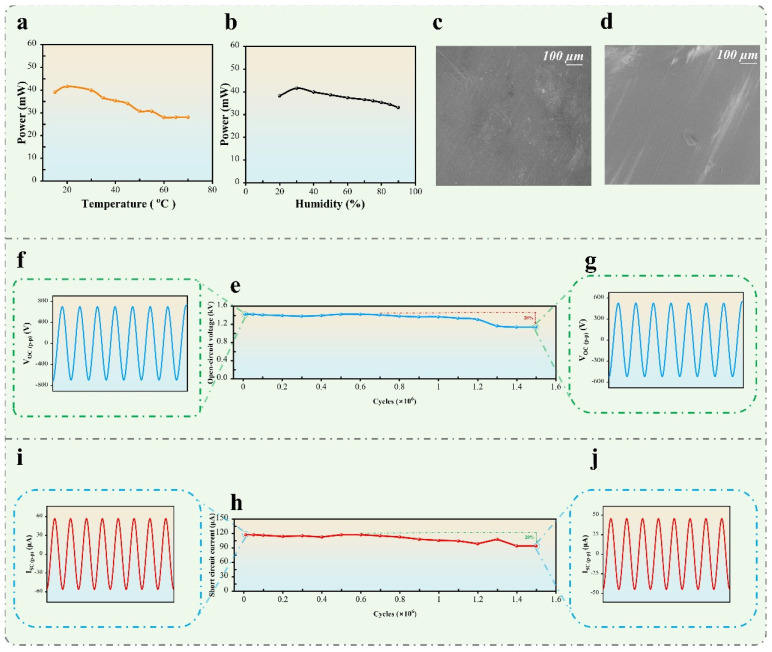
The durability and stability performance of SHLR-TENG under various environmental conditions: (**a**,**b**) the output power of the SHLR-TENG is evaluated under different temperatures and humidity levels, respectively; (**c**,**d**) a comparison of scanning electron microscope (SEM) images of the PI (polyimide) surface before and after 1.5 million cycles; (**e**) the open-circuit voltage under 1.5 million operation cycles; (**f**,**g**) the open-circuit voltage waveform before and after the operation cycles; (**h**) the short-circuit current of the SHLR-TENG over 1.5 million cycles; (**i**,**j**) the short-circuit current waveform before and after the operation cycles.

The SHLR-TENG’s durability in this research has been rigorously evaluated, demonstrating remarkable reliability and efficiency after 1.5 million cycles of constant operation. The capacity of TENGs to maintain a constant electrical output for long periods of time is a basic requirement for assessing their reliability. Additionally, it is well established that environmental elements like humidity and temperature have a significant impact on how well TENGs work. As shown in Figure 8a, charge loss can occur when the temperature range is between 0 and 70 °C; at a high temperature, the electron thermionic emission action is intensified. On the other hand, the power performance is reduced when humidity is between 20 and 90% RH, as high humidity allows water molecules to interact with the electrode surface, as shown in Figure 8b. These aspects are essential in assessing the stability, efficacy, and long-term reliability of TENG systems. Together with other environmental factors, this current research investigation used a scanning electron microscope (SEM) to examine the polyimide (PI) interface before and after 1.5 million cycles, depicted in Figure 8c,d, demonstrating significant alterations in surface morphology. These alterations indicate that mechanical wear, however slight, can influence the output capacity of the triboelectric layer over time. Despite the difficulty posed by performance loss due to device wear, the structural design employed in this work has markedly enhanced the output stability of the SHLR-TENG.

Additionally, Figure 8h,e illustrate that after 1.5 million cycles, the voltage and current waveforms demonstrate alterations, with the output voltage declining from 710 V (as shown in Figure 8f) to approximately 568 V (as shown in Figure 8g), and the current 58.5 µA (as shown in Figure 8i) to 46.8 µA (as shown in Figure 8j). This minor decline, indicating around a 20% variation in output, underscores the system’s resilience and its capacity to effectively transform mechanical energy into electrical energy over prolonged periods with negligible deterioration. The SHLR-TENG system has remarkable long-term stability, maintaining a reasonably consistent output despite slight declines, rendering it an attractive option for continuous energy-harvesting applications.

Furthermore, the SHLR-TENG developed in this study demonstrates exceptional performance and significant advancements compared to existing works in the field. Charge, current, and energy density are crucial for assessing the performance of TENG applications because they directly influence the device’s ability to generate, store, and deliver electrical energy. Charge represents the total amount of electrical energy generated, while current measures how quickly the energy is delivered, both of which are vital for applications requiring fast power transfer. Energy density, on the other hand, demonstrates the amount of energy that is stored in a specific volume or area, which is essential for optimizing the TENG’s compactness and long-term efficiency. Together, these parameters help in fine-tuning the device to meet the specific power and energy needs of various applications. As illustrated in Supplementary Figure S10a–c, the value of the charge density is 5.32 µC·m^−2^, current density is 3.325 mA·m^−2^, and energy density is 37.34 mJ·m^−2^. 

The SHLR-TENG’s surface charge density levels can potentially be approximated using a simple calculation that takes into consideration *V_OC_* and surface charge density. The SHLR-TENG’s theoretical peak-to-peak value of *V_OC_* (*V_OC,P-P_*) has been defined as follows [55]:

(9) $$V_{OC,p-p}=V_{E1}-V_{E2}=\frac{4d.\sigma }{\varepsilon_{0}\varepsilon_{r}}$$

where *σ* represents the surface charge density of the PI film, *ε*_0_ is the permittivity of free space, *d* is the thickness of the PI film, and *ε_r_* is the relative permittivity of the PI. To derive this equation, the following assumptions are applied: the PI film’s thickness is much smaller than the width of the electrodes, and the contact region between the rotator and stator is modeled as a parallel-plate capacitor, with the edge (fringing) effect excluded [55]. According to Equation (9), the calculated *σ* of the PI layer in the SHLR-TENG is 110 µC.m^−2^ at the observed *V_OC_,_P-P_* value of 1420, as illustrated in Supplementary Figure S10d. Equation (9) is applicable solely for illustrating the trend of the V_OC_ (see Supplementary Note S2).

Additionally, power density per unit area is determined by dividing the highest power output by the area throughout which the power is disseminated, depicted in Supplementary Figure S10e. The maximum value attained for PI is 2.36 W·m^−2^·Hz^−1^.

On the other hand, velocity has a significant effect on output power in the system, as shown in Supplementary Figure S12. Initially, as velocity increases, the output power also increases, demonstrating a positive correlation between the two. However, beyond a certain point, further increases in velocity lead to a decrease in power output. This decline in power, despite an increase in velocity, suggests the existence of an optimal operating range where the system performs most efficiently. The Figure illustrates this non-linear relationship, highlighting the point at which further increases in velocity no longer contribute to improved performance and may even result in a reduction in output power [10].

Conversely, the volume of the SHLR-TENG, as depicted in Supplementary Figure S8, has been calculated to be 111.86 cm^3^. Therefore, the power density per unit volume is 371.2 W·m^−3^·Hz^−1^. The results presented underscore SHLR-TENG’s remarkable efficacy among energy-harvesting TENGs. A comprehensive comparison with various TENGs is shown in Supplementary Figure S11 and Supplementary Table S2. These metrics collectively emphasize the superior performance of this TENG, making it a highly competitive and promising solution for a potential option for energy-harvesting applications, particularly in scenarios requiring high power and energy output with compact designs.

Generally, the energy-conversion efficiency of the system was calculated by comparing the mechanical input power delivered by the stepper motor to the electrical output power generated by the triboelectric nanogenerator (TENG). The stepper motor operated at 100 RPM with a torque of 1.2 Nm, resulting in an input power of approximately 1.93 W for each 6.5 cm of linear displacement. The TENG produced a peak voltage of 710 V and a peak current of 58.5 µA, corresponding to an RMS output power of approximately 0.083 W. Based on these values, the energy-conversion efficiency of the system was determined to be 1.076% as shows in Equation (10). This result aligns with typical efficiency values reported for TENG devices and validates the experimental setup as a reliable platform for evaluating TENG performance under controlled mechanical input. With the output power and the input power values, the efficiency of the system was determined using the following formula:

(10) $$Efficiency\;\left(\%\right)=\left(\frac{P_{out}}{P_{in}}\right)\times 100\;\approx \;\left(\frac{0.0208}{1.93}\right)=1.076\%$$

Thus, the efficiency of the system is approximately 1.076%, indicating that only 1.076% of the input power is converted into useful output by the TENG device, with the remainder being lost to system inefficiencies. Supplementary Note S8 shows further details on power calculations of the input and output in this system.

### 3.5. Demonstrations of SHLR-TENG Applications

**Figure 9:**
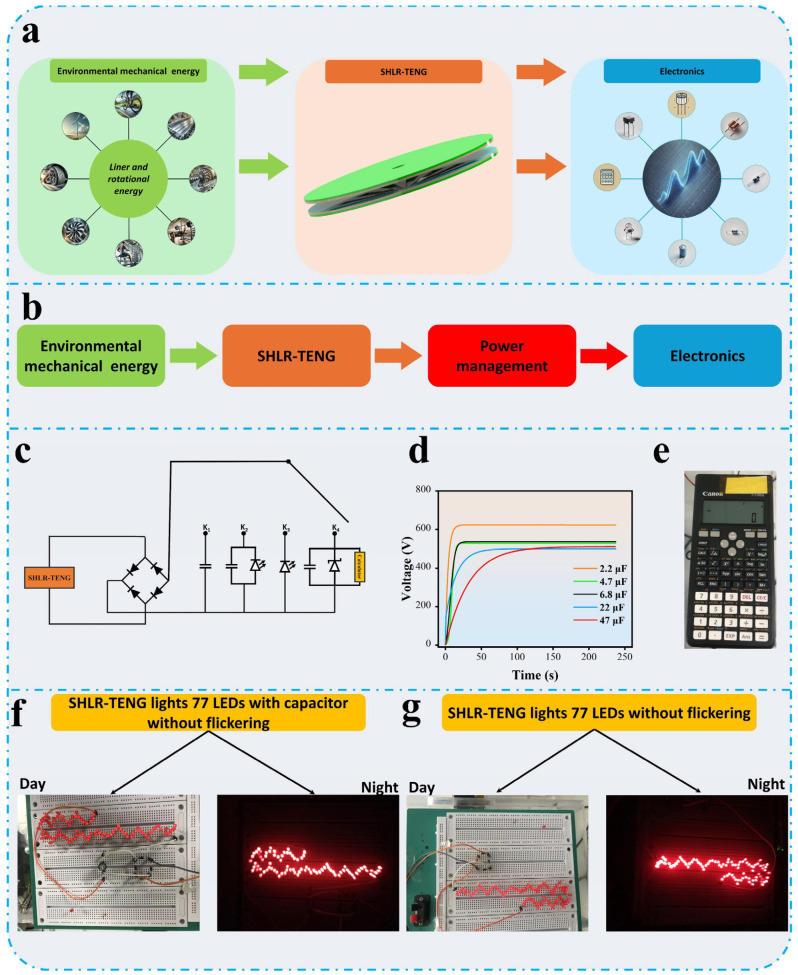
Examples of the utilization of SHLR-TENG: (**a**) a diagram illustrating SHLR-TENG powering electronics directly, (**b**) a schematic emphasizing the efficient use of SHLR-TENG through power management, (**c**) circuit configurations that enable an efficient SHLR-TENG, (**d**) voltage measurements across various capacitors, (**e**) a calculator powered by SHLR-TENG, (**f**) a photo of 77 LEDs powered directly by SHLR-TENG without flickering at a rotation frequency of 1 Hz with a capacitor, and (**g**) without capacitors.

The SHLR-TENG has the potential to function as an independent power source by effectively converting environmental mechanical energy into electrical energy. Its ability to generate a stable sinewave ensures consistent current and voltage output, making it suitable for powering various commercial electronic devices, as illustrated in Figure 9a. The design of the SHLR-TENG is highly versatile, allowing it to harness energy from different types of linear motion, including both rotational and sliding movements. Additionally, its straightforward manufacturing process enhances its applicability in a wide range of real-world scenarios.

To further validate its energy-conversion capabilities, the SHLR-TENG was integrated with a power-management circuit, demonstrating its efficiency in harvesting, storing, and regulating energy. This system enables the SHLR-TENG to supply power to various electronic devices by effectively managing energy flow, as shown in Figure 9b. The circuit connection is depicted in Figure 9c. As shown in Figure 9d, when the circuit switch is connected to K1, the SHLR-TENG successfully charges capacitors of different values (2.2 µF, 4.7 µF, 6.8 µF, 22 µF, and 47 µF), showcasing its ability to store energy for later use. Similarly, when the switch is connected to K2, the stored energy is utilized to power LEDs, as depicted in Figure 9e and Supplementary Video S1.

Furthermore, the SHLR-TENG demonstrates its capability to directly power multiple light-emitting diodes (LEDs). When the switch was connected to K3, the device was able to illuminate 77 LEDs without any flickering, as presented in Supplementary Video S2, either through direct energy transfer or via stored energy, as shown in Figure 9f,g. On the other hand, when the switch connects with K4, it demonstrates that SHLR-TENG was able to power a calculator as shown in Supplementary Video S3. This highlights the device’s ability to provide stable and reliable power output, even without an intermediate storage component. The power-management circuit plays a crucial role in optimizing energy utilization, ensuring that the SHLR-TENG operates efficiently in real-world applications. Its ability to consistently generate and regulate power makes it a viable solution for harvesting mechanical energy from sources such as motion and vibration. This makes it a viable alternative for harvesting energy from mechanical sources such as motion or vibration.

The SHLR-TENG system can be integrated with smart speed bumps for efficient energy harvesting. When the smart bump is subjected to mechanical force, as shown in Supplementary Figure S9, the SHLR-TENG can convert the induced vibrations and pressure into electrical energy. By connecting the SHLR-TENG with the smart bump, it becomes possible to capture energy from vehicles moving over the bump, providing a sustainable source of power for smart city applications. This energy-harvesting system not only enhances the functionality of speed bumps but also contributes to the development of energy-efficient infrastructure.

## 4. Conclusions

This study comprehensively examines the SHLR-TENG under various operating conditions, focusing on resistance load, capacitor load, and their impacts on electrical output. By analyzing the stator–rotor gap and its role in energy-conversion efficiency and material selection, our findings provide valuable insights into optimizing the device’s performance.

A key contribution of this work is the integration of a radially distributed TENG with a transmission mechanism, significantly extending its operational lifespan. Additionally, the disk-based SHLR-TENG, utilizing a gear system to convert linear mechanical energy into rotational energy, ensures a stable, fluctuation-free voltage output. This design enables direct LED powering without requiring energy storage, as a single TENG unit generates a complete alternating-current cycle.

Polymer material (PM) selection played a crucial role in performance optimization, with polyimide (PI) demonstrating the highest efficiency among tested negative PMs, establishing it as the reference material for future comparisons. The SHLR-TENG achieved an average power output of 41.535 mW, a stable open-circuit voltage (V_OC_) of 710 V, and a short-circuit current (I_SC_) of 58.5 µA under identical forward velocities. Even after 1.5 million cycles, the device maintained functionality with only a 20% output reduction, highlighting its durability. The SHLR-TENG also exhibited a surface charge density of 110 µC/m^2^, a power per unit area of 2.36 W·m^−2^·Hz^−1^, and a total power output per volume of 371.2 W·m^−3^·Hz^−1^, underscoring its efficiency in a compact design.

This research is the first to achieve stable voltage generation from horizontal linear motion. The findings emphasize the importance of matching capacitor voltage and resistance to optimize power generation under varying load conditions. By advancing power-management strategies and self-charging power systems, this study presents an effective approach for maximizing average power output. With its outstanding electrical properties, durability, and efficiency, the SHLR-TENG presents a promising solution for powering compact electronic devices, including capacitors and commercial LEDs, driving further advancements in energy-harvesting technologies.

## Data Availability

The data that supports the findings of this study are available from the corresponding author, upon reasonable request.

## Supplementary Materials

The following supporting information can be downloaded at: https://www.mdpi.com/article/10.3390/mi16070801/s1, Figure S1: a (ⅰ,ⅱ) two dimensional of designing rotator and stator. b(ⅰ) Surface images of the rotator. Cu of 19 sectors is patterned onto the surface of the rotator. b(ⅱ) Surface images of the stator. Cu of 38 sectors is patterned onto the surface of the stator; Figure S2: Schematic illustration of the gear system and detailed parameters such as a diameter and number of teeth; Figure S3: the three-dimensional graphic of the gears system; Figure S4: The pictorial images of (a) top, (b) front, (c) back, and (d) right side of the device; Figure S5: Schematic illustration of a cross-sectional view of charge distribution in open-circuit condition at the intermediate state; Figure S6: Schematic illustration of a cross-sectional view of charge distribution in open-circuit condition at the initial state; Figure S7: The circuit diagram of TENG charging capacitor; Figure S8: Three- dimensional of the stator and rotator; Figure S9: Schematic with the smart speed bump energy harvesting SHLR-TENG system, (a) vehicle movement on the bumper, (b) mechanical force, and (c) electrical output; Figure S10: Comparison of the output performance for SHLR-TENG (a) current density, (b) energy density, (c) charge density, (d)surface charge density, (e) power density per area, and (f) power density per volume; Figure S11: Power density of the SHLR-TENG with other TENGs; Figure S12: The relation between power and velocity; Table S1: Show the details of the gears speed; Table S2: The comparison of power density and volume of previously reported TENGs for energy harvesting; Note S1: Gear configuration and energy transfer, optimization of energy transfer and calculations of speeds and ratios; Note S2: Theoretical analysis of operating process in open-circuit condition; Note S3: Theoretical analysis of operating process in short-current condition; Note S4: Formula derivation of stored energy and electric quantity of the capacitor; Note S5: Electrical model of TENG.; Note S6: Average power comparison of TENGs; Note S7: Power management of CV-TENG; Note S8: Calculation of Input Power and system efficiency; Video S1: The red commercial LEDs lit up continuously with storing energy by SHLR-TENG; Video S2: The red commercial LEDs lit up continuously without storage energy by SHLR-TENG; Video S3: The calculator power on continuously without storage energy by SHLR-TENG. References [9,10,11,12,56,57,58,59,60,61,62,63,64,65,66,67,68] are cited in the supplementary materials.
