# Supplementary material for: Constant High-Voltage Triboelectric Nanogenerator with Stable AC for Sustainable Energy Harvesting
Source: Micromachines (Basel). 2025 Jul 9;16(7):801. doi: 10.3390/mi16070801 (PMC12299868; doi:10.3390/mi16070801)
Supplement: Supplementary file 1 [file micromachines-16-00801-s001.zip › SUPPORTING INFORMATION..pdf]

Supporting Information

Constant High-Voltage Triboelectric Nanogenerator with Stable AC for Sustainable Energy Harvesting

Aso Ali Abdalmohammed Shateri[1,2], Salar K. Fatah[2], Fengling Zhuo[1], Nazifi Sani Shuaibu[1], Chuanrui Chen[1], Rui Wan[1], Xiaozhi Wang[1]*

1. Zhejiang University College of Information Science and Electronic Engineering, Hangzhou 310027, Zhejiang Province, China.
2. Department of physics, College of education, University of Garmian, Kalar 46021-KRG - IRAQ.

Supplementary Figure S1 | a (i,ii) two dimensional of designing rotator and stator. b(i) Surface images of the rotator. Cu of 19 sectors is patterned onto the surface of the rotator. b(ii) Surface images of the stator. Cu of 38 sectors is patterned onto the surface of the stator..4

Supplementary Figure S2 | Schematic illustration of the gear system and detailed parameters such as a diameter and number of teeth. 5

Supplementary Figure S3 | the three-dimensional graphic of the gears system. 5

Supplementary Figure S4 | The pictorial images of (a) top, (b) front, (c) back, and (d) right side of the device. 6

Supplementary Figure S5 | Schematic illustration of a cross-sectional view of charge distribution in open-circuit condition at the intermediate state. 6

Supplementary Figure S6 | Schematic illustration of a cross-sectional view of charge distribution in open-circuit condition at the initial state. 7

Supplementary Figure S7 | The circuit diagram of TENG charging capacitor. 16

Supplementary Figure S8 | Three- dimensional of the stator and rotator.................22

Supplementary Figure S9 | Schematic with the smart speed bump energy harvesting SHLR-TENG system, (a) vehicle movement on the bumper, (b) mechanical force, and (c) electrical output. 22

Supplementary Figure S10 | Comparison of the output performance for SHLR-TENG (a) current density, (b) energy density, (c) charge density, (d)surface charge density, (e) power density per area, and (f) power density per volume...23

Supplementary Figure S11 | Power density of the SHLR-TENG with other TENGs. 23

Supplementary Figure S12 | The relation between power and velocity...................24

Supplementary Note S1 | Gear configuration and energy transfer, optimization of energy transfer and calculations of speeds and ratios. 7

Supplementary Note S2 | Theoretical analysis of operating process in open-circuit condition. 10

Supplementary Note S3 | Theoretical analysis of operating process in short-current condition. 13

Supplementary Note S4 | Formula derivation of stored energy and electric quantity of the capacitor. 15

Supplementary Note S5 | Electrical model of TENG. 16

Supplementary Note S6 | Average power comparison of TENGs. 18

Supplementary Note S7 | Power management of CV-TENG. 19

Supplementary Note S8 | Calculation of Input Power and system efficiency........22

Supplementary Table S1 | Show the details of the gears speed...............................10

Supplementary Table S2 | The comparison of power density and volume of previously reported TENGs for energy harvesting. 28

Supplementary Video S1 | The red commercial LEDs lit up continuously with storing energy by SHLR-TENG. 30

Supplementary Video S2 | The red commercial LEDs lit up continuously without storage energy by SHLR-TENG. 30

Supplementary Video S3 | The calculator power on continuously without storage energy by SHLR-TENG. 30

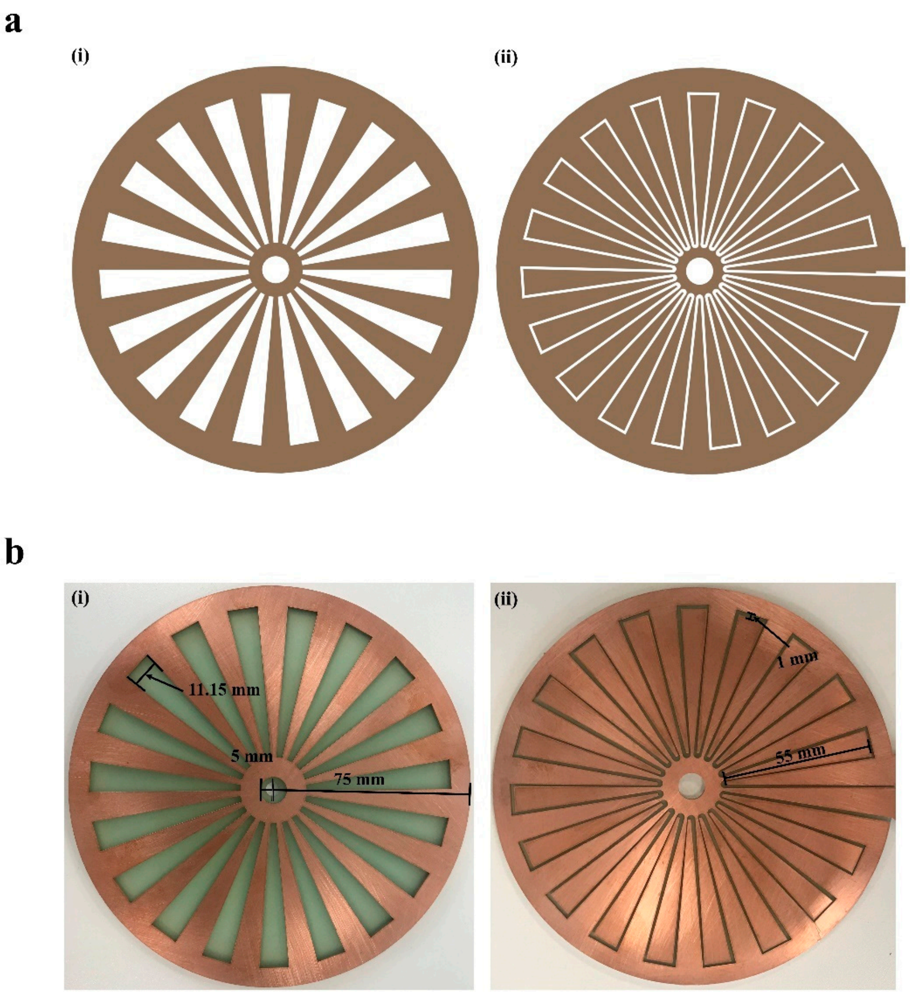

Supplementary Figure S1 | a (i,ii) two dimensional of designing rotator and stator. b(i) Surface images of the rotator. Cu of 19 sectors is patterned onto the surface of the rotator. b(ii) Surface images of the stator. Cu of 38 sectors is patterned onto the surface of the stator.

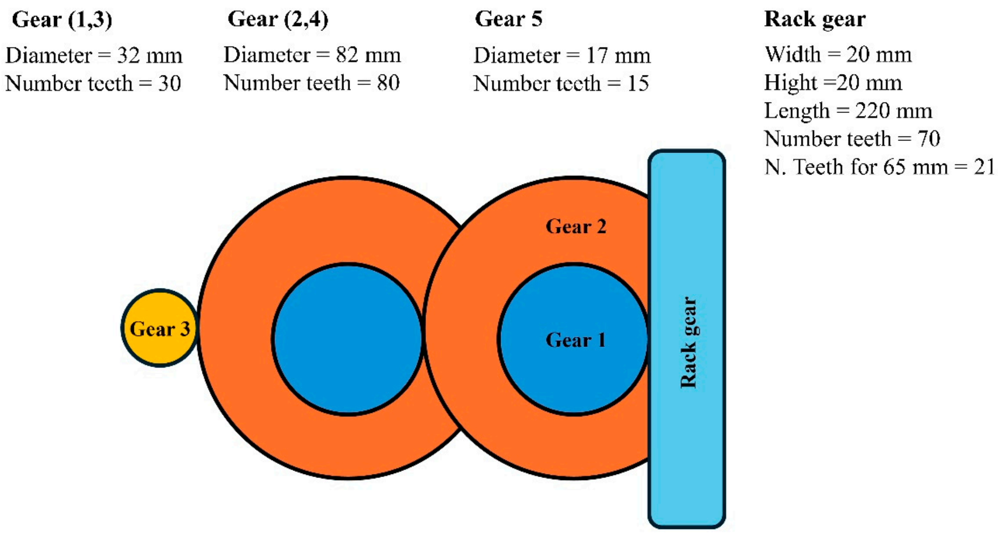

Supplementary Figure S2 | Schematic illustration of the gear system and detailed parameters such as a diameter and number of teeth.

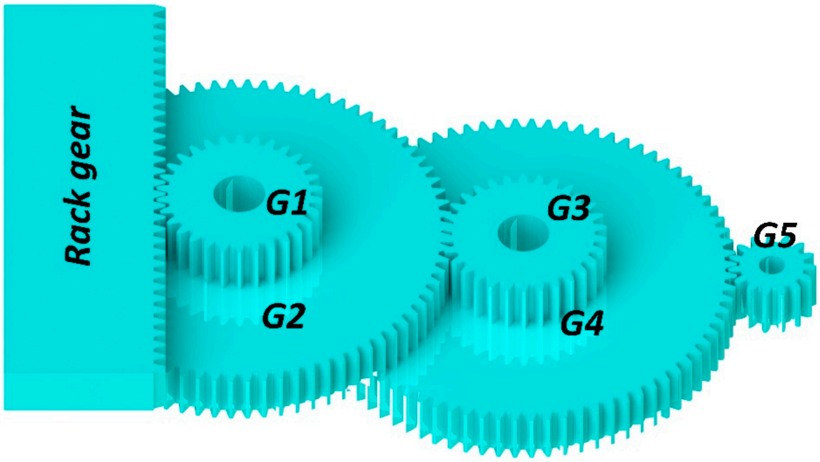

Supplementary Figure S3 | the three-dimensional graphic of the gears system.

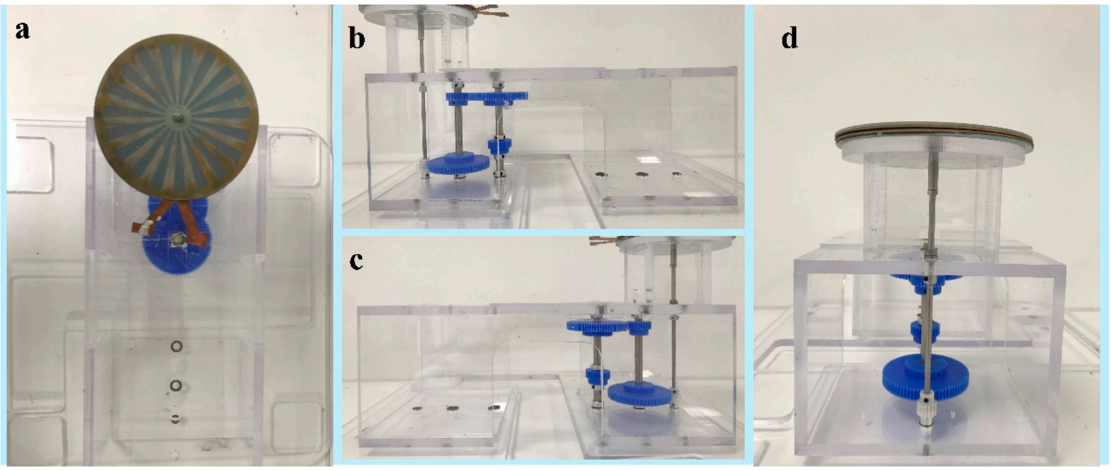

Supplementary Figure S4 | The pictorial images of (a) top, (b) front, (c) back, and (d) right side of the device.

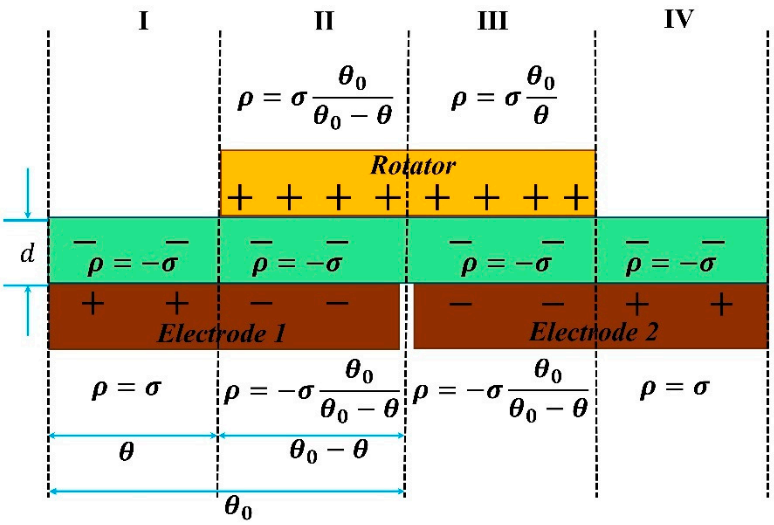

Supplementary Figure S5 | Schematic illustration of a cross-sectional view of charge distribution in open-circuit condition at the intermediate state.

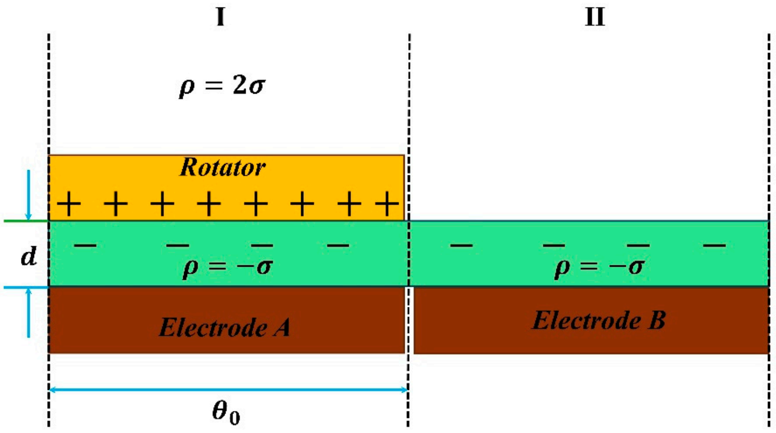

Supplementary Figure S6 | Schematic illustration of a cross-sectional view of charge distribution in open-circuit condition at the initial state.

Supplementary Note S1 | Gear configuration and energy transfer, optimization of energy transfer and calculations of speeds and ratios.

Gear configuration and energy transfer

Our gear system utilizes a multi-stage configuration to efficiently convert linear motion from the rack gear into rotational motion at the output shaft. The specific gear ratios were carefully selected to balance speed and torque, ensuring optimal performance under different speeds and forces.

The system begins with an initial gear reduction from the rack gear to Gear 1. This stage reduces the high-speed linear motion of the rack and increases torque, providing a mechanical advantage. The intermediate gear stages, including Gears 2 and 3, further refine the speed and torque output. The final stage, a gear amplification from Gear 4 to

Gear 5, significantly increases the speed of the output while reducing torque, making the system suitable for applications requiring rapid motion transfer.

Optimization of Energy Transfer

To minimize energy loss and ensure efficiency, the gear system incorporates the following measures:

Material Selection: Nylon was chosen for the gears due to its self-lubricating properties, lightweight design, and low noise generation. Nylon also offers advantages such as corrosion resistance and ease of manufacturing, making it ideal for low-load and low-speed applications.

Calculations of Speeds and Ratios

We have calculated the gear speeds and ratios to further illustrate the energy transfer process. The gear system includes a rack gear and five rotational gears with the following dimensions as shown in Supplementary Figure S5:

Rack Gear: Length = 8 cm, Teeth = 26, Linear Speed = 2 cm/s

Gear 1: Teeth = 30, **Pitch Diameter** = 32 cm

Gear 2: Teeth = 80, **Pitch Diameter** = 82 cm

Gear 3: Teeth = 30, **Pitch Diameter** = 32 cm

Gear 4: Teeth = 80, **Pitch Diameter** = 82 cm

Gear 5: Teeth = 15, **Pitch Diameter** = 17 cm

Step-by-Step Calculations

Rack to Gear 1: Gear Ratio: 21/30

Speed of Gear 1: $\frac{21}{30} \times 2$ =1.4 cm/s

Gear 2 (Same Shaft as Gear 1):

Rotational Speed = 1.4 cm/s

Gear 3 (Driven by Gear 2):

Gear Ratio: 2

Speed: 1.4 cm/s ×2.66 = 3.738 cm/s

Gear 4 (Same Shaft as Gear 3):

Gear 5 (Driven by Gear 4):

Gear Ratio: 4

Speed: 3.724×5.34 = 19.93 cm/s

Supplementary Table S1 | Show the details of the gears speed.

Rack Gear Speed (cm/s)	Gear 1 Speed (cm/s)	Gear 2 Speed (cm/s)	Gear 3 Speed (cm/s)	Gear 4 Speed (cm/s)	Gear 5 Speed (cm/s)
2	1.4	1.4	3.738	3.738	19.93
3	2.1	2.1	5.607	5.607	29.91
4	2.8	2.8	7.476	7.476	39.89
8	5.6	5.6	14.95	14.95	79.74
10	7	7	18.69	18.69	99.76

Supplementary Note S2 | Theoretical analysis of operating process in open-circuit condition.

Based on the assumption that the thickness of the dielectric layer (PMs) is far smaller than its width feature, a simplified model can be used in which any overlapped region between the rotator and the electrodes can be treated as a parallel-plate capacitor without consideration of edge effect. With triboelectric charge density of $-\sigma$ on the PMs surface, the non-overlapped regions on electrode 1 and electrode 2 (regions 1 and 4 in Supplementary Figure S5, respectively) present an induced charge density of $-\sigma$. Given that the net charges on both electrodes should be zero in open circuit condition, the induced charge density on overlapped regions (regions 2 and 3) can be expressed as

Overlapped part on electrode 1 (region 2): $\rho = -\sigma \frac{\theta_0}{\theta_0 - \theta}$ (S1)

Overlapped part on electrode 2 (region 3): $\rho = -\sigma \frac{\theta_0}{\theta_0 - \theta}$ (S2)

Where θ_0 refers to the central angle of the rotator unit, α refers to rotation angle away from the initial position between 0 and θ_0 .

Based on the law of charge conservation, the charge density on different regions of the rotator can be expressed as

Region 2 of the rotator: $\rho = \sigma + \sigma \frac{\theta_0}{\theta_0 - \theta}$ (S3)

Region 3 of the rotator: $\rho = \sigma + \sigma \frac{\theta_0}{\theta_0 - \theta}$ (S4)

Using the charge density shown in Supplementary Figure 5 and Gauss Theorem, the electric field within the dielectric layer for region 2 and 3 can be respectively given as,

$$E_{Region2} = -\frac{\sigma}{\varepsilon_0 \varepsilon_r} \cdot \frac{\theta}{\theta_0 - \theta} \tag{S5}$$

$$E_{Region3} = -\frac{\sigma}{\varepsilon_0 \varepsilon_r} \cdot \frac{\theta_0 - \theta}{\theta} \tag{S6}$$

where ε_r is the relative permittivity of dielectric layer.

Then the potential difference between the rotator and electrode 1(E_1) as well as the potential difference between the rotator and electrode 2 (E_2) can be respectively calculated as,

$$V_{Rotator} - V_{E1} = \frac{d \cdot \sigma}{\varepsilon_0 \varepsilon_r} \cdot \frac{\theta}{\theta_0 - \theta} \tag{S7}$$

$$V_{Rotator} - V_{E2} = \frac{d \cdot \sigma}{\varepsilon_0 \varepsilon_r} \cdot \frac{\theta_0 - \theta}{\theta} \tag{S8}$$

where d is the thickness of the dielectric layer.

Since the rotator made of metal is an equipotential body, the potential difference between the two electrodes (i.e. open-circuit voltage) can be theoretically expressed as

$$V_{OC}(\theta) = V_{E1} - V_{E2} = \frac{d\cdot\sigma}{\varepsilon_0\varepsilon_r}\cdot\frac{\theta}{\theta_0-\theta} - \frac{d\cdot\sigma}{\varepsilon_0\varepsilon_r}\cdot\frac{\theta_0-\theta}{\theta} \qquad \text{(S9)}$$

which is,

$$V_{OC}(\theta) = U_{E1} - U_{E2} = \frac{d\cdot\sigma}{\varepsilon_0\varepsilon_r}\left(\frac{\theta}{\theta_0-\theta} - \frac{\theta_0-\theta}{\theta}\right) \qquad \text{(S10)}$$

However, the above Equation (S10) is not applicable when approaches either 0 or θ_0 . When θ approaches 0, the V_{OC} obtained by this equation goes to positive infinity. This is because when α has a very small value, the rotator only has a very small overlapped area (region 3 in Supplementary Figure S5) with E_2. In this case, the assumption of parallel-plate capacitor does not hold any more. Therefore, deviation occurs. Similarly, when α approaches θ_0, the overlapped area between the rotator and E_1 (region 2 in Supplementary Figure S5) is so small that the basic assumption of parallel-plate capacitor also no longer holds, resulting in negative infinite value of V_{OC} from equation S (10). Therefore, equation (S10) is only used to illustrate the changing trend of the V_{OC} when the rotator spins. In order to calculate the V_{OC} at the initial and final positions, the following derivation based on electrostatics is used.

At region 1 on the left (Supplementary Figure S5), the net triboelectric charge at the contact interface is σ , while the net triboelectric charge is – σ at region 2 on the right. Based on the model of infinitely large plane with uniform charging, the electric potential of E_1 and E_2 with an infinitely far position as a zero-potential reference point can be respectively calculated by

$$V_{\mathrm{E1}} = \frac{d \cdot \sigma}{\varepsilon_0 \varepsilon_r} \tag{S11}$$

$$V_{\mathrm{E2}} = -\frac{d \cdot \sigma}{\varepsilon_0 \varepsilon_r} \tag{S12}$$

Therefore, the V_{OC} at the initial state is

$$V_{OC(inirial)} = V_{\mathrm{E1}} - V_{\mathrm{E2}} = \frac{2d \cdot \sigma}{\varepsilon_0 \varepsilon_r} \tag{S13}$$

Based on the same reasoning, the $\mathrm{V_{OC}}$ at the final state is

$$V_{OC(final)} = V_{E1} - V_{E2} = -\frac{2d \cdot \sigma}{\varepsilon_0 \varepsilon_r} \tag{S14}$$

Consequently, the peak-to-peak value of the $\mathrm{V_{OC}}$ is

$$V_{p-p} = V_{E1} - V_{E2} = \frac{4d \cdot \sigma}{\varepsilon_0 \varepsilon_r} \tag{S15}$$

Supplementary Note S3 | Theoretical analysis of operating process in short-current condition.

Based on the model of volume-changing capacitors, we can assume a voltage ($V_{E1\,E2}$)-charge ($Q_{E1\,E2}$) relationship between E_1 and E_2 as follows,

$$V_{E1\,E2} = -\frac{1}{C_{E1\,E2}} \times Q_{E1\,E2} + V_{OC} \tag{S16}$$

where $C_{E1\,E2}$ is the capacitance between E_1 and E_2.

$C_{E1\,E2}$ can be treated as a series connection of two capacitors, which are the capacitor formed by the rotator and E1 and the capacitor formed by the rotator and E2:

$$C_{\mathrm{E1\,E2}} = \frac{1}{\frac{1}{C_{Rotator-E1}} + \frac{1}{C_{Rotator-E2}}} \quad \text{(S17)}$$

$$C_{Rotator-E1} = \frac{\varepsilon_0 \varepsilon_r P(\theta_0 - \theta)}{d} \quad \text{(S18)}$$

$$C_{Rotator-E2} = \frac{\varepsilon_0 \varepsilon_r P(\theta)}{d} \quad \text{(S19)}$$

where $P(\theta_0 - \theta)$ is the overlapped area between the rotator and E1, and $P(\theta)$ is the overlapped area between the rotator and E2.

Finally, we can get

$$V_{E1E2} = -\left[\frac{d \cdot \theta_0}{\varepsilon_0 \varepsilon_r \cdot \theta} \cdot \frac{360^o}{\theta_0 - \theta} \cdot \frac{1}{\pi(r_2^2 - r_1^2)}\right] \times Q_{E1\,E2} + \frac{d \cdot \sigma}{\varepsilon_0 \varepsilon_r}\left(\frac{\theta}{\theta_0 - \theta} - \frac{\theta_0 - \theta}{\theta}\right) \quad \text{(S20)}$$

where r_2 is the outer radius of the rotator, and r_1 is the inner radius of the rotator. In short circuit condition, $V_{E1,E2} = 0$. Therefore, charge transferred between the two electrodes in short circuit condition is,

$$Q_{E1\,E2} = \frac{2\theta_0 - \theta}{360^o} \cdot \sigma \cdot \pi(r_2^2 - r_1^2) \quad \text{(S21)}$$

By submitting $\theta = 0$ and $\theta_0 = \theta$ into Equation (S20), we can obtain the total charge that transport as the rotator spins from $\theta = 0$ to $\theta_0 = \theta$ by the following equation

$$Q = \frac{2\theta_0}{360^o} \cdot \sigma \cdot \pi(r_2^2 - r_1^2) \quad \text{(S22)}$$

Supplementary Note S4 | Formula derivation of stored energy and electric quantity of the capacitor.

Formula derivation of stored energy and electric quantity of the capacitor. The total energy stored in the capacitor (E^C) and the total charge stored in the capacitor (Q^C) can be given by:

$$E^C = \int_0^t Pdt = \int_0^t CU_C \frac{dU_C}{dt} dt = \int_0^t CU_C \, dU_C = \frac{1}{2} CU_C^2 \quad \text{(S23)}$$

$$Q^C = CU_C \quad \text{(S24)}$$

where *P* is the instantaneous power of capacitor, *C* is the capacitance of the capacitor, U_C represents the voltage of the capacitor. Due to the voltage of the capacitor is a time-varying variable, E^C and Q^C are going to change over time. Energy flowing to the capacitor per cycle and charge flowing to the capacitor per cycle can be derived below:

$$E_n^C = \frac{1}{2} CU_C (n)^2 - \frac{1}{2} CU_C (n-1)^2 \quad \text{(S25)}$$

$$Q_n^C = CU_C (n) - CU_C (n-1) \quad \text{(S26)}$$

where n is the cycle number, V(n) is the final voltage value of *n* cycle.

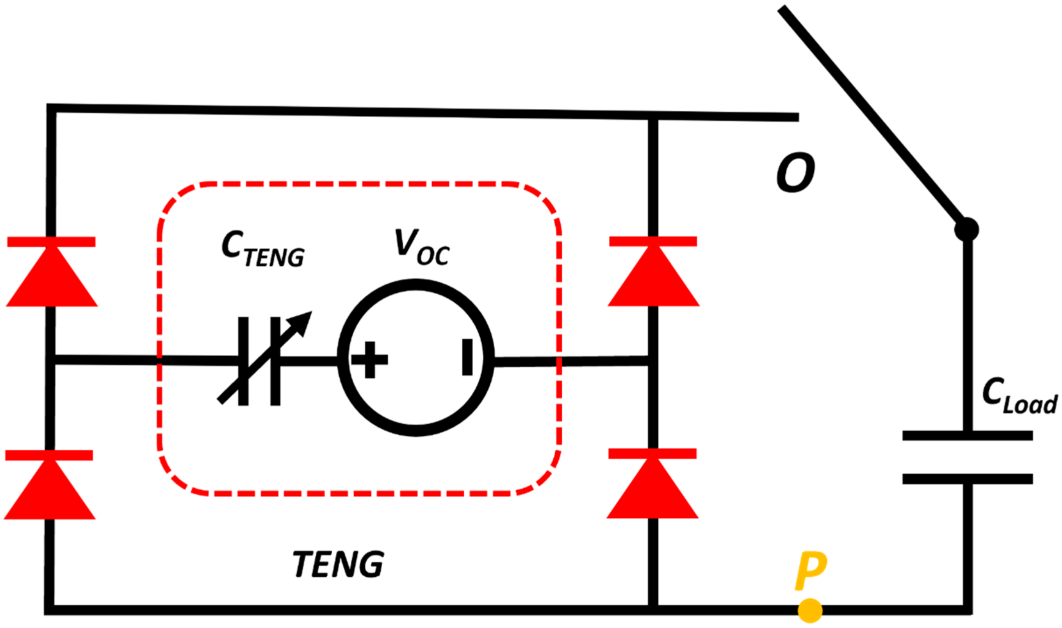

Supplementary Figure S7 │The circuit diagram of TENG charging capacitor.

Supplementary Note S5│Electrical model of TENG.

As the model established by Niu et al [1] Define C_T is the variable capacitor and C_L is capacitance load of TENG, where C_{min} and C_{max} are the minimum and maximum value of variable capacitance of TENG, respectively. It is believed that the charge through node P conforms to the law of charge conservation according to Kirchhoff's law as shown in Fig. S. Before charging starts, the initial charge on C_T and C_L are zero. The charge on node P is also zero. The mathematical expression is $Q_1^P = 0$, and Q_n^P represents the total charge on node P at the beginning of the nth cycle. The charge (Q_T and Q_L) stored in the capacitor (C_T and C_L) at the end of the first half period in the nth cycle is as follow:

$$Q_{n,1end}^T = C_L \frac{Q_{SC,max} + Q_n^P}{C_L + C_{min}} \quad \text{(S27)}$$

$$Q_{n,1end}^L = C_L \frac{C_L Q_{SC,max} + C_{min} Q_n^P}{C_L + C_{min}} \quad \text{(S28)}$$

where $Q_{n,1end}^T$ represents the value at the end of the first half period in the nth cycle. From half period of the first cycle to the beginning of half period of the second cycle,

due to the full wave rectifier circuit, the polarity of C_L will be reversed. At the beginning of the second half period of the *n*th cycle, the charge stored in node *P* ($Q_{n\,mid}^{P}$) can be given by the following equation:

$$Q_{n,mid}^{P} = -Q_{n,2begin} + Q_{n,2begin}^{C} = -Q_{n,1end} + Q_{n,1end}^{C} \quad \text{(S29)}$$

The second half cycle is also a one-way charging process. At the end of the second half cycle, the charges stored on C_T and C_L (Q_T and Q_L) can be given by the following equation:

$$Q_{n,2end} = -\frac{C_{max} Q_{n,mid}^{P}}{C_L + C_{max}} \quad \text{(S30)}$$

$$Q_{n,2end}^{C} = -\frac{C_{max}\, Q_{n,mid}^{P}}{C_L + C_{max}} \quad \text{(S31)}$$

At the end of the second half cycle, the relative polarity of C_L with respect to the TENG is reversed again. Therefore, the charge ($Q_{n}^{P}{}_{+1}$) on the node *P* at the time of the *n+1* cycle is given by the following equation:

$$Q_{n+1}^{P} = -Q_{n+1,1begin} + Q_{n+1,1begin}^{C} = -Q_{n,2end} + Q_{n,2end}^{C} \quad \text{(S32)}$$

By this formula up here, the recursion of Q_n^P can be expressed as

$$Q_{n+1}^{P} = \frac{C_L - C_{max}}{C_L + C_{max}} \frac{C_L - C_{min}}{C_L + C_{min}} Q_n^P + 2\frac{C_L - C_{max}}{C_L + C_{max}} \frac{C_L}{C_L - C_{min}} Q_{SC,max} \quad \text{(S33)}$$

With the boundary condition of $Q_1^P = 0$, the above recursion can be solved as follows:

$$Q_n^P = \frac{C_L - C_{max}}{C_{min} + C_{max}} Q_{SC,max} - \frac{C_L - C_{max}}{C_{min} + C_{max}} Q_{SC,max} \left[\frac{(C_L - C_{max})(C_L - C_{min})}{(C_L + C_{max})(C_L + C_{min})}\right]^{n-1} \quad \text{(S34)}$$

Therefore, after the first *n* charging cycle, the voltage $|V_{n\ ,2end}^{C}|$on the C_L can be expressed as

$$\left|V_{n,2\mathrm{end}}^{C}\right| = \frac{Q_{SC,max}}{C_{min}+C_{max}}\left(1-\left[1-\frac{2(C_{min}+C_{max})C_L}{(C_L+C_{max})(C_L+C_{min})}\right]^n\right) \qquad \text{(S35)}$$

In practical applications, C_L is generally much larger than C_{min} and C_{max}. Using this condition, it can be further simplified as

$$\left|V_{n,2\mathrm{end}}^{C}\right| = \frac{Q_{SC,max}}{C_{min}+C_{max}}\left(1-e^{-\frac{2(C_{min}+C_{max})\,n}{C_L}}\right) = \frac{Q_{SC,max}}{C_{min}+C_{max}}\left(1-e^{-\frac{2(C_{min}+C_{max})\,ft}{C_L}}\right) \text{(S36)}$$

It can be seen from the above formula that the case can be equal to a voltage source with internal resistance charging the same capacitor, and they all follow the same exponential saturation trend. The voltage value can be described as follows:

$$V_{sat} = \lim_{n\to\infty}\left|V_{n,2\mathrm{end}}^{C}\right| = \frac{Q_{SC,max}}{C_{min}+C_{max}} \qquad \text{(S37)}$$

Like the first-order RC charging circuit, independent of C_L, V_{sat} is only a function of the parameters of the TENG.

In the real-time charging behavior of TENG, it is necessary to consider whether the voltage of TENG is higher than the voltage of capacitor when the transferred charge of TENG flowing to the capacitor.

Supplementary Note S6 | Average power comparison of TENGs.

Average power comparison of TENGs. Triboelectric nanogenerator (TENG) plays an increasingly significant role in distributed power sources or self-powered sensors with its remarkable superiority of low cost, easy fabrication, diverse choice of materials, and high efficiency at low operation frequency. Despite average power density is a key character to compare different TENGs fairly, rare research focuses on the standard for TENG as distributed power source. Considering disordered mechanical energy input and a variety of different design styles of TENG, a general-purpose standard of average power is expected to evaluate TENGs as distributed power sources. Here, volume

specific average power density per Hertz (Wm^{-3} Hz^{-1}) is proposed as a standard for comparison of average power of different TENGs as distributed power sources. Several works based on phase shift design are compared with the same standard as shown in Supplementary Table S2. It is easy to acquire the device parameter in the style of cylindrical structure TENG, while it is hard to obtain the height information of planar device because researches pay more attention on charge density every square meter. Therefore, area specific average power density per Hertz (Wm^{-2} Hz^{-1}) is also proposed as a standard for fair comparison in this paper. Meanwhile, previous research has reported the mass specific average power (Wkg^{-1}), refrence1 it is also another suitable standard for fair comparison in terms of considering the mass in practical application of TENG. Therefore, mass specific average power density per Hertz ($W\ kg^{-1}$ Hz^{-1}) also could be considered as a standard for comparison of average power of different TENGs as distributed power sources in the future.

Supplementary Note S7 | Power management of CV-TENG.

From formula (S14), SHLR-TENG can be regard as a constant voltage source with internal resistance as discussed in Note S2. Therefore, the electrical model of SHLR-TENG could be equivalent as the first-order RC charging circuit as shown in Fig. S17. Define the voltage of SHLR-TENG is U, the inherent resistance of SHLR-TENG is R, the capacitor is C, the voltage of inherent resistance is U_R, and the voltage of capacitor is U_C. When the switch S is not closed, there is no voltage at both ends of the capacitor, that is, U_C is equal to 0, and the current (i) in the circuit is 0. In the transient process from the moment the switch S is closed to the capacitor voltage is U_C, the following equation can be listed for the circuit according to Kirchhoff's law：

$$U = U_R + U_C = iR + U_C \qquad (36)$$

The charge stored in capacitor is given by the following equation：

$$Q = CU_C \qquad (37)$$

Substitute Equation (37) into the current definition of capacitor as follows：

$$i = \frac{dq}{dt} = \frac{dU_C}{dt} = C\frac{dU_C}{dt} \qquad (38)$$

Equation (S38) is obtained when the reference directions of UC and i are consistent. It shows that the capacitor current is proportional to the change rate of the capacitor applied voltage.

Substitute Equation (S38) into Equation (S36) as follows：

$$RC\frac{dU_C}{dt} + U_C = U \qquad (S39)$$

Equation (S39) is a first-order linear differential equation with constant coefficients, it could be solved to obtain the variation rule of capacitor voltage as follows:

$$U_C = U\left(1 - e^{-\frac{t}{RC}}\right) \qquad (S40)$$

The changing law of circuit current is as follows:

$$i = \frac{U}{R}\left(e^{-\frac{t}{RC}}\right) \qquad (S41)$$

The voltage of the resistance changes as follows:

$$u_R = iR = R\frac{U}{R}\left(e^{-\frac{t}{RC}}\right) = U\left(e^{-\frac{t}{RC}}\right) \qquad (S42)$$

From the above formula, during the charging process, the voltage of capacitor increases exponentially with time. After a period of time, the capacitor voltage is equal to the electromotive force of the SHLR-TENG. While the current of circuit and the voltage of resistance decrease exponentially with time to zero.

The instantaneous power P_C and energy E_C of the capacitor is as follows:

$$P_C = U_C i = CU_C \frac{dU_C}{dt} \qquad \text{(S43)}$$

$$E_C = \int_0^t Pdt = \int_0^t CU_C \frac{dU_C}{dt}\, dt = \int_0^t CU_C\ dU_C = \frac{1}{2}\, CU_C^2 \qquad \text{(S44)}$$

The charge stored in the capacitor is moved by the electromotive force of the power supply, so the electrical energy E from the power supply is as follow:

$$E = Uq = UCU_C \qquad \text{(S45)}$$

Therefore, the charging efficiency up to time t is as follows:

$$\eta = \frac{E_C}{E} = \frac{CU_C^2}{2UCU_C} = \frac{1}{2}\left(1 - e^{\frac{-t}{RC}}\right) \qquad \text{(S46)}$$

The energy-output efficiency presents very low at the initial stage of charging because the voltage of capacitor is low, and the energy output does not increase much. Even the energy output efficiency is gradually elevated as time goes by, the maximum energy-output efficiency is only 50%, which is consistent with previous report from V-Q plot.

Supplementary Note S8 | Calculation of Input Power and system efficiency

To calculate the input power required by the stepper motor, we need to relate the rotational motion of the motor to the linear displacement of the system. Given that the motor produces a 6.5 cm (0.065 m) linear travel and operates at a speed of 100 RPM (Revolutions Per Minute), we can calculate the power based on the torque generated by the motor.

Step 1: Calculate the Circumference of the Pulley

The stepper motor drives a pulley with a radius of 31.83 mm (0.03183 m). The linear displacement per revolution is equal to the circumference of the pulley, calculated as:

$$C = 2\pi r = 2\pi \times 0.03183\, m \approx 0.200\, m$$

Thus, for each revolution of the motor, the belt moves 0.200 meters.

Step 2: Calculate the Number of Revolutions per Minute

At 100 RPM, the motor completes 100 revolutions per minute, so the total linear displacement per minute is:

Distance per minute = 100×0.200 m =20 m

This means the system can move 20 meters of linear distance per minute.

Step 3: Calculate the Required Power

The torque generated by the stepper motor is 1.2 Nm (Newton-meters). We can calculate the angular velocity (ω) using the RPM:

$$\omega = \frac{2\pi\ RPM}{60} = \frac{2\pi\ \times 100}{60} \approx 10.47\ rad/s$$

The input power (Pin) required by the system can then be calculated using the formula:

$$P = \tau \cdot \omega = 1.2\ N.m\ \times 10.47 \frac{rad}{s} \approx 12.57\ W$$

Step 4: Calculate Power for 6.5 cm Linear Travel

Given that the system moves 6.5 cm (0.065 meters) per revolution, we now calculate the power needed specifically for 6.5 cm of linear travel. The total linear distance per minute was found to be 20 meters, so to find the power for just 6.5 cm of travel, we scale the power accordingly:

$$Power\ per\ 6.5\ cm = \frac{P_{in}}{total\ distance\ per\ minute} \times 0.065\ m$$

$$= \frac{12.57\ W}{20\ m} \times 0.065\ m\ \approx 1.93\ W$$

Thus, the input power required for 6.5 cm of linear travel is approximately 1.93 watts.

Efficiency Calculation of the Triboelectric Nanogenerator (TENG) System

In this study, a stepper motor is used to drive a system connected to a Triboelectric Nanogenerator (TENG) device. The motor provides rotational power, which is then converted into linear motion to drive the TENG. The input power required for the motor to generate a linear displacement of 6.5 cm per rotation was calculated as 1.93 watts. This represents the power consumed by the motor to convert rotational motion into linear movement.

The TENG device generates output power based on its peak-to-peak voltage and current values. The peak voltage of the TENG device is 710 V, and the peak-to-peak current is 58.5 μA. To calculate the output power, the peak voltage and current values were first converted into their respective RMS values, which are used in power calculations:

$$V_{rms} = \frac{V_P}{\sqrt{2}} \approx \frac{710}{\sqrt{2}} = 502.0458\, V$$

$$I_{rms} = \frac{I_P}{\sqrt{2}} \approx \frac{58.2}{\sqrt{2}} = 41.3657 \times 10^{-6}\, A$$

The output power of the TENG was then calculated using the formula:

$$P_{out} = V_{rms} \times I_{rms} \approx 502.0458\ \times 41.3657 = 0.020767499\, W$$

With the output power and the input power values, the efficiency of the system was determined using the following formula:

$$Efficiency\ (\%) = \left(\frac{P_{out}}{P_{in}}\right) \times 100\ \approx\ \left(\frac{0.0208}{1.93}\right) = 1.076\%$$

Thus, the efficiency of the system is approximately 1.076%, indicating that only 1.076% of the input power is converted into useful output by the TENG device, with the remainder being lost to system inefficiencies.

Discussion on Energy Conversion Efficiency

It is common for energy conversion systems, including those based on Triboelectric Nanogenerators (TENGs), to exhibit relatively low efficiency, particularly in the early

stages of development. This is primarily due to several inherent challenges in the energy conversion process, including friction, material limitations, and energy losses during mechanical-to-electrical conversion. For instance, while TENGs efficiently convert mechanical energy into electrical energy, there are still significant losses due to factors like friction, heat dissipation, and imperfect charge transfer.

Furthermore, the materials used in TENGs (such as certain polymers) may not have optimal triboelectric properties, leading to less efficient energy conversion. The mechanical design, including the alignment and interaction between triboelectric materials, also plays a significant role in efficiency. In real-world applications, energy conversion systems often suffer from suboptimal design, such as poor contact between materials or inefficient actuation mechanisms, which results in energy losses.

The typical efficiency range for TENGs falls between 1% to 10%, though some high-performance systems have achieved efficiencies closer to 20%-30% in specific configurations. This low efficiency is expected and is part of the challenge of developing energy harvesting technologies. Despite these limitations, TENGs are still promising for applications where low-power, sustainable energy harvesting is required, such as in wearable devices and sensor networks.

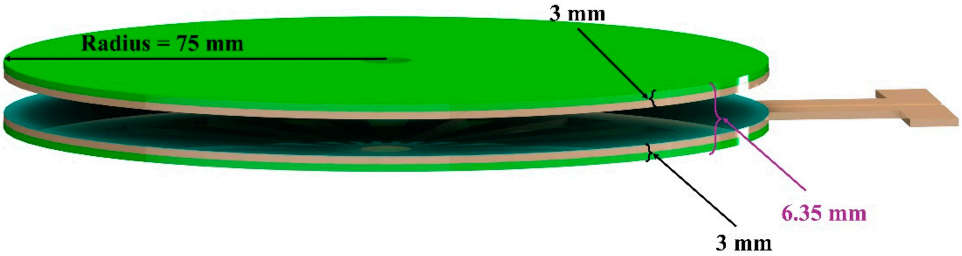

Supplementary Figure S8 | Three- dimensional of the stator and rotator.

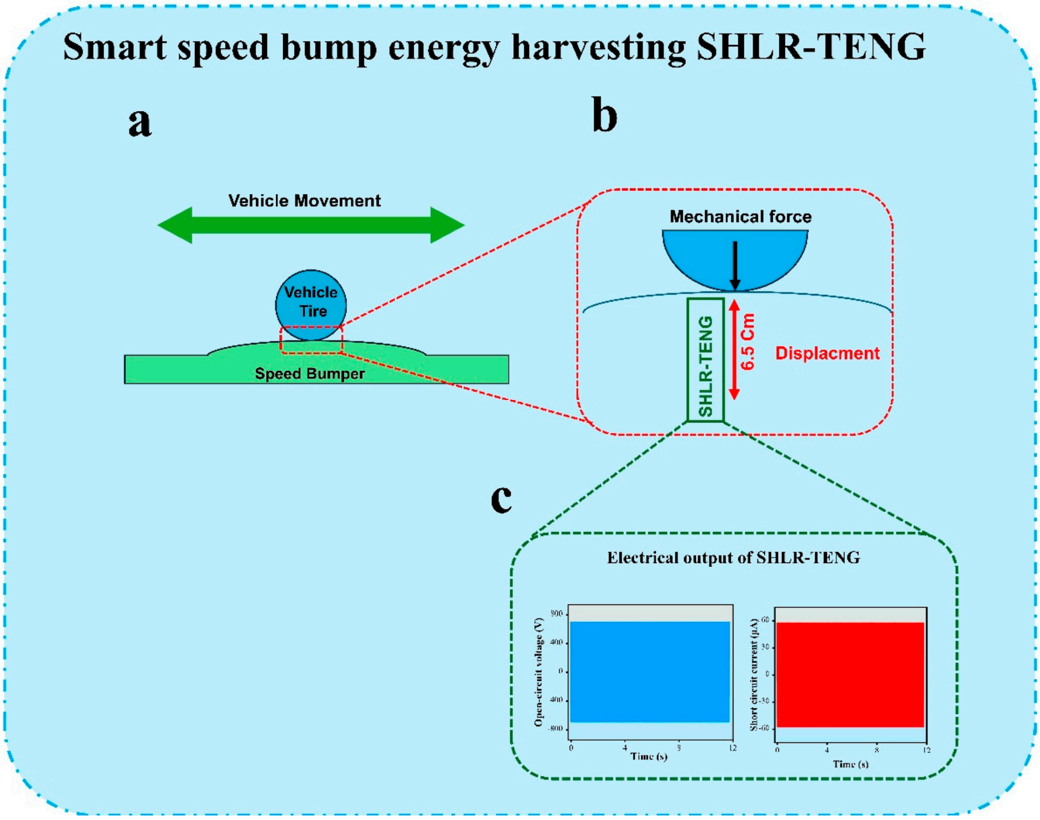

Supplementary Figure S9 | Schematic with the smart speed bump energy harvesting SHLR-TENG system, (a) vehicle movement on the bumper, (b) mechanical force, and (c) electrical output.

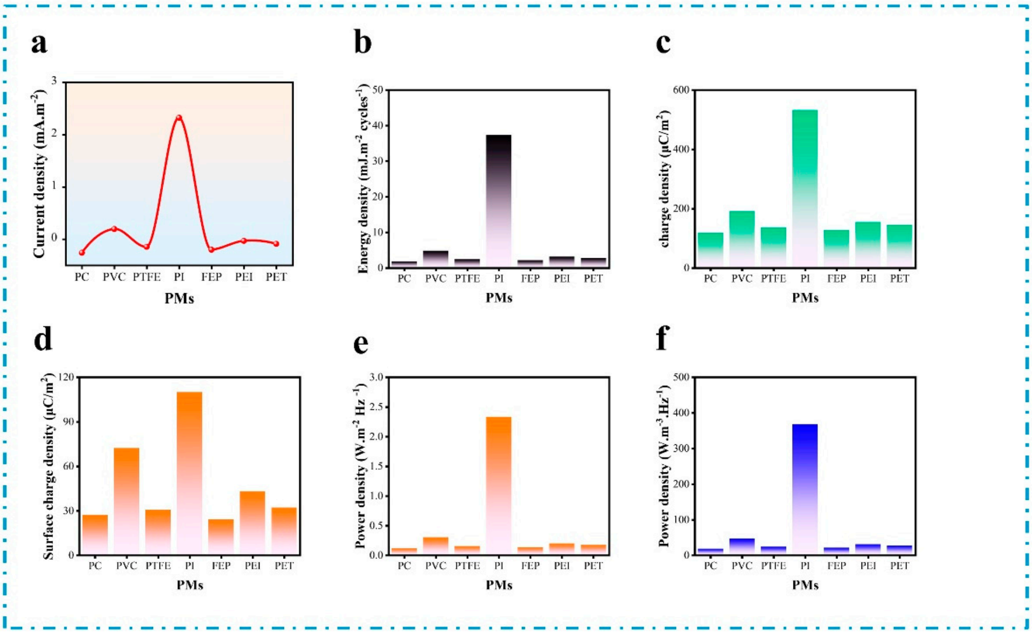

Supplementary Figure S10 | Comparison of the output performance for SHLR-TENG (a) current density, (b) energy density, (c) charge density, (d)surface charge density, (e) power density per area, and (f) power density per volume.

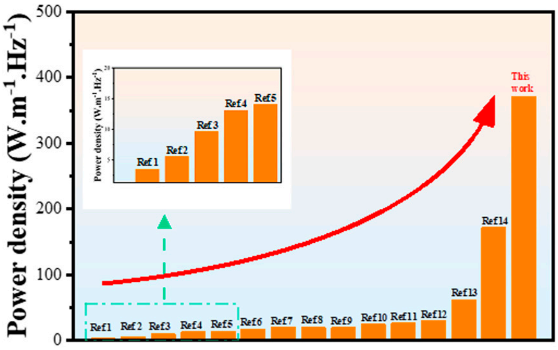

Supplementary Figure S11 | Power density of the SHLR-TENG with other TENGs.

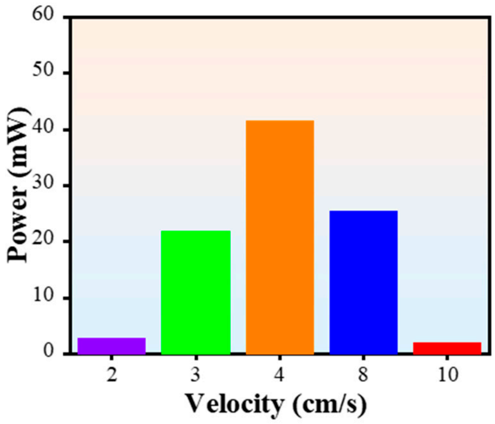

Supplementary Figure S12 | The relation between power and velocity

Supplementary Table S2 | The comparison of power density and volume of previously reported TENGs for energy harvesting.

	Year	Name	Working Mode	Application				
				Power (mW)	Power Density ($W/m^3 \cdot Hz$)	Optimal Resistance	Volume (cm^3)	Ref.
1	2025	SHLR-TENG	Sliding Mode	41.535	371.2 $W.m^{-3}.Hz^{-1}$	12 MΩ	111.86	This work
2	2024	DHLR-TENG	Sliding Mode	Peak: 33.27 (Motor, 1Hz)	171.616	9 MΩ	193.865	[2]
3	2023	SR-TENG	Rolling mode	Peak: 16 (Motor, 2 Hz)	Peak: 26.435 (Motor, 2 Hz)	300 MΩ (Motor, 2 Hz)	302.63	[3]
4	2023	FE-TENG	Rolling mode	N/A	Peak: 5.6 (Motor, 1.25 Hz)	1000 MΩ (Motor, 1.25 Hz)	N/A	[4]

5	2019	Self-assembly TENG	Rolling mode	**Peak: 8.75** (Motor, 1.67 Hz)	**Peak: 19.55** (Motor, 1.67 Hz)	**1000 MΩ** (Motor, 1.67 Hz)	**268**	[5]
6	2023	Chiral Network of Triboelectric Nanogenerator	Rolling mode	**Peak: 7.88** (motor, 1.5 Hz)	**Peak: 19.61** (motor, 1.5 Hz)	**1000 MΩ** (Motor, 1 Hz)	**267.95**	[6]
7	2021	Spherical TENG	Rolling mode	**Peak: 10.7** (Motor, 5.9 Hz)	**Peak: 3.47** (Motor, 5.9 Hz)	**300 MΩ** (Motor, 5.9 Hz)	**527**	[7]
8	2019	T-TENG	Rolling mode	**Peak: 0.143** (Motor, 1.2 Hz)	**Peak: 1.03** (Motor, 1.2 Hz)	**2000 MΩ** (Motor, 1.2 Hz)	**1390**	[8]
9	2021	S-TENG	Rolling mode	**Peak: 25.22** (Motor, 2 Hz)	**Peak: 17.325** (Motor, 2 Hz)	**500 MΩ** (Motor, 2 Hz)	**727.85**	[9]
10	2022	S-TENG	Rolling mode	**N/A**	**Peak: 24.535** (Motor, 2 Hz)	**300 MΩ** (Motor, 2 Hz)	**N/A**	[10]
11	2021	WT-TENG	Sliding Mode	**N/A**	**Peak: 13.1** (Motor, 1 Hz)	**Peak: 10 GΩ** (Motor, 1 Hz)	**5.2**	[11]
12	2020	CS-TENG	Contact-Separation	**Peak: 126.8** (Motor, 1 Hz)	**Peak: 17.81** (Motor, 1 Hz)	**517 kΩ** (Motor, 0.7 Hz)	**488.79**	[12]
13	2019	OB-TENG	Contact-Separation	**Peak: 38.7** (Motor, 1 Hz)	**Peak: 9.675** (Motor, 1 Hz)	**13.8 MΩ** (Motor, 1 Hz)	**4000**	[13]
14	2023	DE-TENG	Sliding Mode	**Peak: 0.85** (Motor, 0.5 Hz)	**Peak: 61.9** (Motor, 0.5 Hz)	**500 MΩ**	**27.3**	[14]
15	2023	CS-TENG	Sliding Mode	**Peak: 14.36** (motor, 1.75 Hz)	**Peak: 19.71** (motor, 1.75 Hz)	**560 kΩ** (Motor, 1.75 Hz)	**416**	[15]

16	2019	TD-TENG	Sliding Mode	**Peak: 29.4** (Motor, 1 Hz)	**Peak: 30.21** (Motor, 1 Hz)	**1 MΩ** (Motor, 1 Hz)	**973.3**	[16]
17	2023	WLM-TENG	Sliding Mode	**Peak: 50** (Wave, 1 Hz)	**Peak: 14.1** (Wave, 1 Hz)	**200 kΩ** (Wave, 1 Hz)	**3546.09**	[17]

Supplementary Video S1 │The red commercial LEDs lit up continuously with storing energy by SHLR-TENG.

Supplementary Video S2│The red commercial LEDs lit up continuously without storage energy by SHLR-TENG.

Supplementary Video S3│ The calculator power on continuously without storage energy by SHLR-TENG.

References

[1] J. Wang, S. Li, F. Yi, Y. Zi, J. Lin, X. Wang, Y. Xu, Z.L. Wang, (2016) Sustainably powering wearable electronics solely by biomechanical energy. Nature Communications 7:12744. https://doi.org/10.1038/ncomms12744.

[2] A.A.A. Shateri, F. Zhuo, N.S. Shuaibu, R. Wan, L. Xu, D. Hazarika, B. Gyawali, X. Wang, (2025) Generating a Full Cycle of Alternative Current Using a Triboelectric Nanogenerator for Energy Harvesting. Micromachines 16:48.

[3] Y. Duan, H. Xu, S. Liu, P. Chen, X. Wang, L. Xu, T. Jiang, Z.L. Wang, (2023) Scalable rolling-structured triboelectric nanogenerator with high power density for water wave energy harvesting toward marine environmental monitoring. Nano Research 16:11646-11652.

[4] Z. Jing, J. Zhang, J. Wang, M. Zhu, X. Wang, T. Cheng, J. Zhu, Z.L. Wang, (2022) 3D fully-enclosed triboelectric nanogenerator with bionic fish-like structure for harvesting hydrokinetic energy. Nano Research 15:5098-5104.

[5] X. Yang, L. Xu, P. Lin, W. Zhong, Y. Bai, J. Luo, J. Chen, Z.L. Wang, (2019) Macroscopic self-assembly network of encapsulated high-performance triboelectric nanogenerators for water wave energy harvesting. Nano Energy 60:404-412.

[6] X. Li, L. Xu, P. Lin, X. Yang, H. Wang, H. Qin, Z.L. Wang, (2023) Three-dimensional chiral networks of triboelectric nanogenerators inspired by metamaterial's structure. Energy & Environmental Science 16:3040-3052.

[7] Z. Yuan, C. Wang, J. Xi, X. Han, J. Li, S.-T. Han, W. Gao, C. Pan, (2021) Spherical triboelectric nanogenerator with dense point contacts for harvesting multidirectional water wave and vibration energy. ACS Energy Letters 6:2809-2816.

[8] M. Xu, T. Zhao, C. Wang, S.L. Zhang, Z. Li, X. Pan, Z.L. Wang, (2019) High power density tower-like triboelectric nanogenerator for harvesting arbitrary directional water wave energy. ACS nano 13:1932-1939.

[9] H. Wang, Z. Fan, T. Zhao, J. Dong, S. Wang, Y. Wang, X. Xiao, C. Liu, X. Pan, Y. Zhao, (2021) Sandwich-like triboelectric nanogenerators integrated self-powered buoy for navigation safety. Nano Energy 84:105920.

[10] H. Wang, C. Zhu, W. Wang, R. Xu, P. Chen, T. Du, T. Xue, Z. Wang, M. Xu, (2022) A stackable triboelectric nanogenerator for wave-driven marine buoys. Nanomaterials 12:594.

[11] H. Wu, Z. Wang, Y. Zi, (2021) Multi-mode water-tube-based triboelectric nanogenerator designed for low-frequency energy harvesting with ultrahigh volumetric charge density. Advanced Energy Materials 11:2100038.

[12] H. Wang, L. Xu, Y. Bai, Z.L. Wang, (2020) Pumping up the charge density of a triboelectric nanogenerator by charge-shuttling. Nature Communications 11:4203.

[13] W. Zhong, L. Xu, X. Yang, W. Tang, J. Shao, B. Chen, Z.L. Wang, (2019) Open-book-like triboelectric nanogenerators based on low-frequency roll–swing oscillators for wave energy harvesting. Nanoscale 11:7199-7208.

[14] X. Liang, S. Liu, S. Lin, H. Yang, T. Jiang, Z.L. Wang, (2023) Liquid–solid triboelectric nanogenerator arrays based on dynamic electric-double-layer for harvesting water wave energy. Advanced Energy Materials 13:2300571.

[15] H. Qiu, H. Wang, L. Xu, M. Zheng, Z.L. Wang, (2023) Brownian motor inspired monodirectional continuous spinning triboelectric nanogenerators for extracting energy from irregular gentle water waves. Energy & Environmental Science 16:473-483.

[16] Y. Bai, L. Xu, C. He, L. Zhu, X. Yang, T. Jiang, J. Nie, W. Zhong, Z.L. Wang, (2019) High-performance triboelectric nanogenerators for self-powered, in-situ and real-time water quality mapping. Nano Energy 66:104117.

[17] J. Han, Y. Liu, Y. Feng, T. Jiang, Z.L. Wang, (2023) Achieving a large driving force on triboelectric nanogenerator by wave-driven linkage mechanism for harvesting blue energy toward marine environment monitoring. Advanced Energy Materials 13:2203219.
